# Interleukin 1β-mediated HOXC10 Overexpression Promotes Hepatocellular Carcinoma Metastasis by Upregulating PDPK1 and VASP

**DOI:** 10.7150/thno.41712

**Published:** 2020-02-19

**Authors:** Yunzhi Dang, Jie Chen, Weibo Feng, Chenyang Qiao, Weili Han, Yongzhan Nie, Kaichun Wu, Daiming Fan, Limin Xia

**Affiliations:** 1 State Key Laboratory of Cancer Biology, National Clinical Research Center for Digestive Diseases and Xijing Hospital of Digestive Diseases, The fourthMilitary Medical University, Xi'an 710032, Shaanxi Province, China.; 2 Department of Radiation Oncology, Xijing Hospital. The fourth Military Medical University, Xi'an, Shaanxi, 710032, China.; 3 Department of Gastroenterology, Tongji Hospital of Tongji Medical College, Huazhong University of Science and Technology, Wuhan 430030, Hubei Province, China.

**Keywords:** hepatocellular carcinoma, metastasis, homeobox C10, interleukin-1β, interleukin 1 receptor type 1.

## Abstract

**Rationale:** Metastasis and recurrence are the primary reasons for the high mortality rate of human hepatocellular carcinoma (HCC) patients. However, the exact mechanism underlying HCC metastasis remains unclear. The Homeobox (HOX) family proteins, which are a highly conserved transcription factor superfamily, play important roles in cancer metastasis. Here, we report a novel role of HOXC10, one of the most upregulated HOX genes in human HCC tissues, in promoting HCC metastasis.

**Methods:** The expression of HOXC10 and its functional targets was detected by immunohistochemistry in two independent human HCC cohorts. Luciferase reporter and chromatin immunoprecipitation assays were used to measure the transcriptional regulation of target genes by HOXC10. The effect of HOXC10-mediated invasion and metastasis were analyzed by Transwell assays and by an orthotopic metastasis model.

**Results:** Elevated expression of HOXC10 was positively correlated with the loss of tumor encapsulation and with higher tumor-nodule-metastasis (TNM) stage and poor prognosis in human HCC. Overexpression of HOXC10 promoted HCC metastasis by upregulating metastasis-related genes, including 3-phosphoinositide-dependent protein kinase 1 (PDPK1) and vasodilator-stimulated phosphoprotein (VASP). Knockdown of PDPK1 and VASP inhibited HOXC10-enhanced HCC metastasis, whereas upregulation of PDPK1 and VASP rescued the decreased metastasis induced by HOXC10 knockdown. Interleukin-1β (IL-1β), which is the ligand of IL-1R1, upregulated HOXC10 expression through the c-Jun NH2-terminal kinase (JNK)/c-Jun pathway. HOXC10 knockdown significantly reduced IL-1β-mediated HCC metastasis. Furthermore, Anakinra, a specific antagonist of IL-1R1, inhibited IL-1β-induced HOXC10 upregulation and HCC metastasis. In human HCC tissues, HOXC10 expression was positively correlated with PDPK1, VASP and IL-1R1 expression, and patients with positive coexpression of HOXC10/PDPK1, HOXC10/VASP or IL-1R1/HOXC10 exhibited the poorest prognosis.

**Conclusions:** Upregulated HOXC10 induced by IL-1β promotes HCC metastasis by transactivating PDPK1 and VASP expression. Thus, our study implicates HOXC10 as a prognostic biomarker, and targeting this pathway may be a promising therapeutic option for the clinical prevention of HCC metastasis.

## Introduction

Hepatocellular carcinoma (HCC), a highly aggressive primary liver cancer, ranks as the fifth most common malignancy worldwide and the second leading cause of cancer-related death in Asia 1. Surgical resection is still the best therapeutic strategy for patients with early disease, but many HCC patients develop postsurgical recurrence or metastasis with poor 5-year survival rates. The high rates of tumor recurrence and distant metastasis after surgical resection are the major reason for the poor prognosis of patients with HCC 2. Therefore, exploring the molecular mechanism underlying HCC metastasis is still eagerly needed.

Homeobox (HOX) genes are a highly conserved subgroup of the homeobox superfamily that play important roles in embryonic development and physiological processes, such as apoptosis, differentiation, receptor signaling, motility and angiogenesis 3. In mammals, 39 HOX genes have been identified and classified in four separate clusters (A, B, C and D) 4. In recent years, mounting evidence has indicated that the deregulation of HOX family genes plays critical roles in cancer initiation and progression 3. For example, overexpression of HOXB7 5, HOXA11 6 and HOXA13 7 promotes cancer invasion and metastasis and indicates poor prognosis. In contrast, HOXA5 and HOXA9 are downregulated in human cancer and function as tumor suppressor genes (TSG) 8-9. These studies indicate that HOX family genes are master regulators of cancer progression and metastasis. In mammals, HOXC10 regulates the cell cycle, mitotic progression, and embryonic development 10. Deregulated HOXC10 functions as an oncogene and contributes to malignant behaviors in several human cancers, including glioma 11-12, cervical squamous cell carcinoma 13 and gastric cancer 14. However, whether HOXC10 is involved in HCC progression and metastasis remains unknown.

Interleukin-1β (IL-1β) is a critical inflammatory cytokine, and constitutive activation of IL-1β plays a crucial role in chronic inflammation 15. Active IL-1β binds to interleukin 1 receptor type I (IL-1R1) and activates nuclear factor kappa-B (NF-κB), c-Jun NH2-terminal kinase (JNK), extracellular signal-regulated protein kinases (ERK1/2), and p38 signaling pathways 16. A recent study showed that IL-1β deficiency strikingly alleviated obesity-induced HCC development 17. Moreover, IL-1β promotes liver tumorigenesis and HCC metastasis by enhancing the transcription of oncogenes such as Gankyrin and hypoxia-inducible factor-1α (HIF-1α) 18-19. Such findings have confirmed the important role of IL-1β in HCC. However, the molecular mechanism underlying IL-1β-mediated HCC metastasis remains unclear.

Here, to study the potential role of HOX family genes in HCC, we investigated their expression in human HCC tissues. Among the 39 HOX genes, homeobox C10 (HOXC10) was found to be the most upregulated HOX gene (Supplementary Figure S1). We demonstrated that HOXC10 promoted HCC metastasis by upregulating 3-phosphoinositide-dependent protein kinase 1 (PDPK1) and vasodilator-stimulated phosphoprotein (VASP). IL-1β-IL-1R1 signaling upregulated HOXC10 expression via the JNK/c-Jun pathway. Anakinra, a specific antagonist for IL-1R1, inhibited IL-1β-induced HOXC10 upregulation and HCC metastasis.

## Materials and Methods

### Patients, follow-up and HCC tissue samples

This study was approved by the Ethics Committee of the Fourth Military Medical University and Tongji Medical College. All patients from the 2 separate centers provided full consent for the study and the consent was written and based on the ethical guidelines of the 1975 Declaration of Helsinki. The number of the approved protocol by the respective bioethics committee was KY20193057 (Approval From the drug clinical trial Ethics Committee, First Affiliated Hospital of Fourth Military Medical University). Cohort I included 397 adult patients with HCC who underwent curative resection between 2006 and 2008 at the Xijing Hospital of Fourth Military Medical University (Xi'an, China). Cohort II included 325 adult patients with HCC who underwent curative resection between 2006 and 2008 at the Tongji Hospital of Tongji Medical College (Wuhan, China). A preoperative clinical diagnosis of HCC was based on the diagnostic criteria of the American Association for the Study of Liver Diseases. The inclusion criteria were as follows: (a) distinctive pathologic diagnosis, (b) no preoperative anticancer treatment or distant metastases, (c) curative liver resection, and (d) complete clinical-pathological and follow-up data. The differentiation statuses were graded according to the method proposed by Edmondson and Steine. The pTNM classification for HCC was based on The American Joint Committee on Cancer/International Union Against Cancer staging system (6th edition, 2002).

The follow-up data for cohorts I and II were summarized at the end of December 2016 (range 4-96 months). The patients were evaluated every 2-3 months during the first 2 years and every 3-6 months thereafter. All follow-up examinations were performed by physicians who were blinded to the study. During each check-up, the patients were monitored for tumor recurrence by assaying serum AFP levels and performing abdominal ultrasound examinations. A computed tomography and/or magnetic resonance imaging examination, together with a chest radiographic examination, was performed every 3-6 months. The diagnostic criteria for HCC recurrence were the same as the preoperative criteria. The time to recurrence and overall survival were the primary endpoints. The time to recurrence was calculated from the date of resection to the date of tumor recurrence diagnosis. The overall survival was calculated from the date of resection to the date of death or last follow-up.

In addition, 20 normal liver tissues and 90 pairs of fresh HCC tissues and adjacent nontumor tissue samples were collected after surgical resection and were used to investigate the mRNA expression levels of HOXC10. Twenty pairs of adjacent nontumor tissues, primary HCC tissues and metastatic HCC tissues were collected after surgical resection and evaluated for protein and mRNA expression of HOXC10, PDPK1, VASP and IL-1R1.

### Immunohistochemistry

HCC specimens and matched adjacent tissues were used to construct a tissue microarray (Shanghai Biochip Co., Ltd. Shanghai, China). The tissue microarray was stained for HOXC10 (abcam, ab153904), PDPK1 (Santa Cruz, sc-17765), VASP (abcam, ab229624) and IL-1R1(abcam, ab154524) expression. The array was scored independently by two pathologists for both the staining intensity and the extent of the protein expression across the section.

Immunohistochemistry was performed on 4-μm-thick, routinely processed paraffin-embedded sections. Briefly, after baking on a panel at 60 °C for an hour, the tissue sections were deparaffinized with xylene and rehydrated through gradient ethanol immersion. Endogenous peroxidase activity was quenched by 3% (vol/vol) hydrogen peroxide in methanol for 12 min, followed by three 3-min washes with phosphate-buffered saline (PBS). Then the slides were immersed in 0.01 mol/L citrate buffer solution (pH 6.0) and placed in a microwave oven for 30 min. After washing in PBS (pH 7.4, 0.01 mol/L), the sections were incubated in a moist chamber at 4 °C overnight with the primary antibody diluted in PBS containing 1% (wt/vol) bovine serum albumin. Negative controls were performed by replacing the primary antibody with preimmune mouse serum. After three 5 min washes with PBS, the sections were treated with a peroxidase-conjugated second antibody (Santa Cruz) for 30 min at room temperature, followed by additional three 5 min washes with PBS. Reaction product was visualized with diaminobenzidine for 2 min. Images were obtained under a light microscope (Olympus, Japan) equipped with a DP70 digital camera.

Analyses were performed by two independent observers who were blinded to the clinical outcome. The immunostaining intensity was scored on a scale of 0 to 3: 0 (negative), 1 (weak), 2 (medium) or 3 (strong). The percentage of positive cells was evaluated on a scale of 0 to 4: 0 (negative), 1 (1%-25%), 2 (26%-50%), 3 (51%-75%), or 4 (76%-100%). The final immuno-activity scores were calculated by multiplying the above two scores, resulting an overall scores which range from 0~12. Each case was ultimately considered “negative” if the final score ranges from 0~3, and “positive” if the final score ranges from 4~12.

### Chromatin immunoprecipitation Assay (ChIP)

Chromatin immunoprecipitation assays were conducted with a Magana CHIP A/G (catalog #17-10085, Merck Millipore). In briefly, HCC cells transfected with lentivirus were crosslinked using 1% formaldehyde for 10 minutes at 37℃. After cell lysis, the DNA was fragmented by sonication. ChIP grade antibody HOXC10, or IgG (negative control) was used to immunoprecipitated the fragment DNA. Then, qRT-PCR was used to amplify the corresponding binding site on the promoters (see Supplementary Table S6 for the primers used).

For ChIP assays of tissues, cells were first separated from six pairs of fresh frozen HCC tissues and normal liver tissues collected after surgical resection. In detail, surgically extracted tumor tissues were first washed by 1×cold, PBS, 5min, for three times and added to medium supplemented with antibiotic and antifungal agents. Use a clean razor blade to cut a pie of tissue (around 5mm^3^) into small piece (typical 1mm^3^ or smaller). Then, digestion the tissues with DNase I (20 mg/mL; Sigma-Aldrich) and collagenase (1.5 mg/mL; Sigma-Aldrich) and placed on table concentrator, 37℃, for 1h. At the end of the hour, we filtered the dissociated cells through 100-μm-pore filters rinsed with fresh media. The 1×red cell lysis was added to the tissues and incubated for 5 minutes to lysis the red blood cell, followed by another rinse. The dissociated cells were crosslinked using 1% formaldehyde for 10 minutes at 37℃. After cell lysis, the DNA was fragmented by sonication. ChIP grade antibody HOXC10, or IgG (negative control) was used to immunoprecipitated the fragment DNA. Then, qRT-PCR was used to amplify the corresponding binding site on the promoters.

### Statistical analysis

All values were recorded as the mean ± standard deviation (sd). P values were statistically analyzed by the χ^2^ test for categorical variables and by Student's test for quantitative data. Survival was calculated with the Kaplan-Meier method (log-rank test). Multivariate analysis was performed by Cox regression analysis. P<0.05 was considered statistically significant. Statistical values were calculated with SPSS software (Version 20.0).

Detailed descriptions of all other materials and methods can be found in the online Supplementary Materials.

## Results

### Elevated expression of HOXC10 promotes HCC metastasis and positively correlates with poor prognosis

Previous studies indicated that abnormal expression of HOX family genes plays important roles in many types of human cancer 3. We sought to identify HOX family members that are deregulated in HCC. Therefore, we analyzed the mRNA expression levels of 39 *HOX* genes in 10 normal liver tissues and 30 pairs of HCC tissues and adjacent nontumor tissues. The expression of *HOXA11*, *HOXB1*, *HOXB8*, *HOXB9*, *HOXB13*, *HOXC5*, *HOXC8*, *HOXC12*, *HOXD11* and *HOXD12* was undetected. The expression levels of *HOXA1*, *HOXA2*, *HOXA4*, *HOXA7*, *HOXA9*, *HOXB2*, *HOXB3*, *HOXB4*, *HOXB6*, *HOXC6*, *HOXC9*, *HOXC11*, *HOXC13*, *HOXD1*, *HOXD3*, *HOXD4*, *HOXD8*, *HOXD10* and *HOXD13* were similar in HCC tissues and adjacent nontumor tissues. The expression level of *HOXA5* was significantly lower in HCC tissues than in adjacent nontumor tissues. In addition, the expression levels of *HOXA3*, *HOXA6*, *HOXA10*, *HOXA13*, *HOXB5*, *HOXB7*, *HOXC4*, *HOXC10* and *HOXD9* were higher in HCC tissues than in adjacent nontumor tissues. Among these 9 *HOX* genes, *HOXC10* was identified as the most upregulated *HOX* gene (Supplementary Figure S1). To further investigate which of the 9 *HOX* genes were essential for the migration and invasion of HCC cells, we knocked down the 9 *HOX* genes individually (Supplementary Figure S2A). Interestingly, Transwell assays indicated that cell migration and invasion were remarkably inhibited by the downregulation of HOXC10 in HCCLM3 cells (Supplementary Figure S2B). Therefore, we focused on the HOXC10 gene for further study.

**Figure 1:**
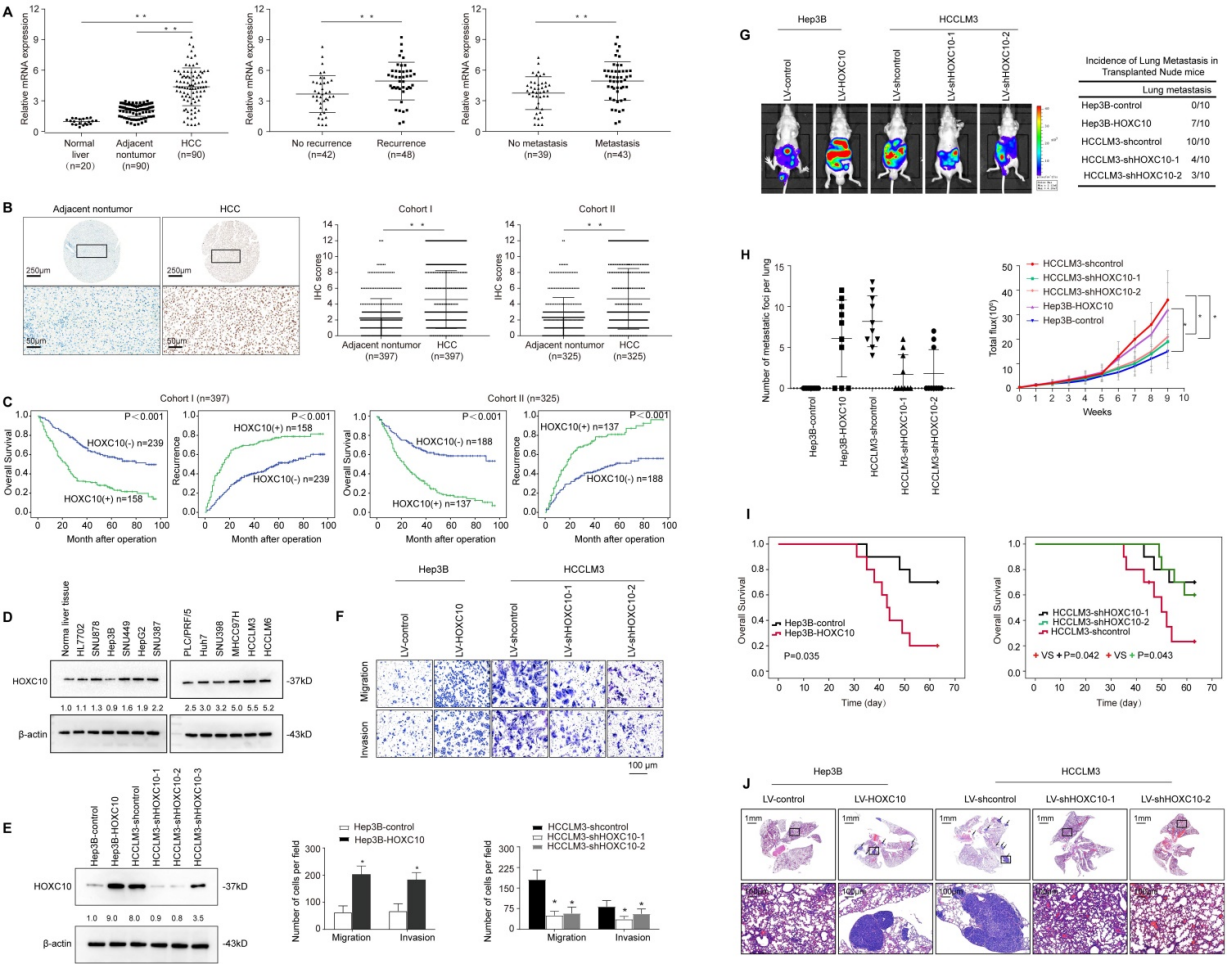
** Elevated HOXC10 expression promotes HCC invasion and metastasis and indicates a poor prognosis in human HCC.** (A) Relative *HOXC10* mRNA expression in 20 normal liver tissues and 90 paired HCC and adjacent nontumorous tissues (left). Relative *HOXC10* mRNA expression in HCC patients with (n=48) or without (n=42) recurrence (middle). Relative *HOXC10* mRNA expression in HCC patients with (n=43) or without (n=39) metastasis (right). (B) Representative images of IHC staining and IHC scores of HOXC10 in human HCC tissues from two independent cohorts of patients. The scale bars represent 250 µm (low magnification) and 50 µm (high magnification). (C) Kaplan-Meier analysis of the correlation of HOXC10 expression with recurrence and overall survival in Cohort I and Cohort II. (D) Western blotting analysis of HOXC10 expression in normal liver tissue and human HCC cell lines. (E) Western blotting analysis of HOXC10 expression in the indicated HCC cells. (F) Transwell assay analysis of the migration and invasion abilities of the indicated HCC cells. (G-J) *In vivo* metastasis assays. The indicated HCC cell lines were transplanted into the livers of nude mice. (G) Bioluminescent images and incidence of lung colonization. (H) Number of lung-colonizing nodules and intensity of bioluminescence signals. (I) Overall survival. (J) Representative HE staining of lung tissues from the different groups is shown (J). The scale bars represent 1 mm (low magnification) and 100 μm (high magnification). All the data are shown as the mean±s.d. * P<0.05 ** P˂0.01.

*HOXC10* mRNA expression levels were analyzed in 90 HCC tissue samples and their matched adjacent nontumor tissue samples and in 20 normal tissue samples by real-time PCR. The mRNA levels of *HOXC10* were dramatically higher in HCC tissues than in adjacent nontumor tissues and normal liver tissues (Figure 1A, left). Notably, levels of HOXC10 were significantly higher in patients with recurrence than in patients without recurrence (Figure 1A, middle). Furthermore, the HOXC10 expression level was much higher in patients with metastasis than in patients without metastasis (Figure 1A, right).

Next, we detected HOXC10 protein levels in two independent tissue microarrays of cohort I (n=397) and cohort II (n=325) by IHC staining (Figure 1B). In both cohorts, HOXC10 expression was dramatically higher in HCC tissues than in adjacent nontumor tissues. In both cohorts, elevated HOXC10 expression was positively correlated with maximal tumor size, tumor encapsulation loss, and higher tumor-nodule-metastasis (TNM) stage (Table 1). HCC patients with positive HOXC10 expression had a significantly higher risk of recurrence and shorter overall survival time than patients with negative HOXC10 expression (Figure 1C). Multivariate analysis indicated that HOXC10 was an independent predictor for both recurrence and survival (Table 2). Taken together, these studies suggest that HOXC10 is a prognostic biomarker in human HCC.

We then examined the expression level of HOXC10 in established human HCC cells and found that HOXC10 expression was higher in HCC cell lines than in normal cell lines and normal liver tissues (Figure 1D). Next, we used three lentivirus shRNA to knockdown HOXC10 expression. Western blot analyses confirmed that endogenous HOXC10 was depleted by LV-shHOXC10-1 and LV-shHOXC10-2 (Figure 1E). Hep3B, SNU878 and HCCLM3 were then used to establish four stable cell lines, Hep3B-HOXC10, SNU878-HOXC10, HCCLM3-shHOXC10-1, and HCCLM3-shHOXC10-2, by lentiviral transduction. The expression of HOXC10 in these two cell lines was analyzed by Western blotting (Figure 1E, Supplementary Figure S5A).

To study the functions of HOXC10 in HCC metastasis, we performed both* in vitro* and *in vivo* experiments. Transwell assays showed that HOXC10 overexpression increased the migration and invasion of Hep3B and SNU878 cells, and that knockdown of HOXC10 resulted in the opposite effects (Figure 1F, Supplementary Figure S5B). An* in vivo* metastasis assay showed that HOXC10 upregulation increased the lung metastasis rate and the number of metastatic lung nodules and decreased the survival time of the nude mice. In contrast, HOXC10 downregulation markedly decreased the lung metastasis rate and the number of metastatic lung nodules and prolonged the survival time of the nude mice (Figure 1G-J, Supplementary Figure S5C-H). These studies suggested that overexpression of HOXC10 promoted HCC invasion and metastasis.

### Metastasis-related genes *PDPK1* and *VASP* are direct transcriptional targets of HOXC10

**Figure 2:**
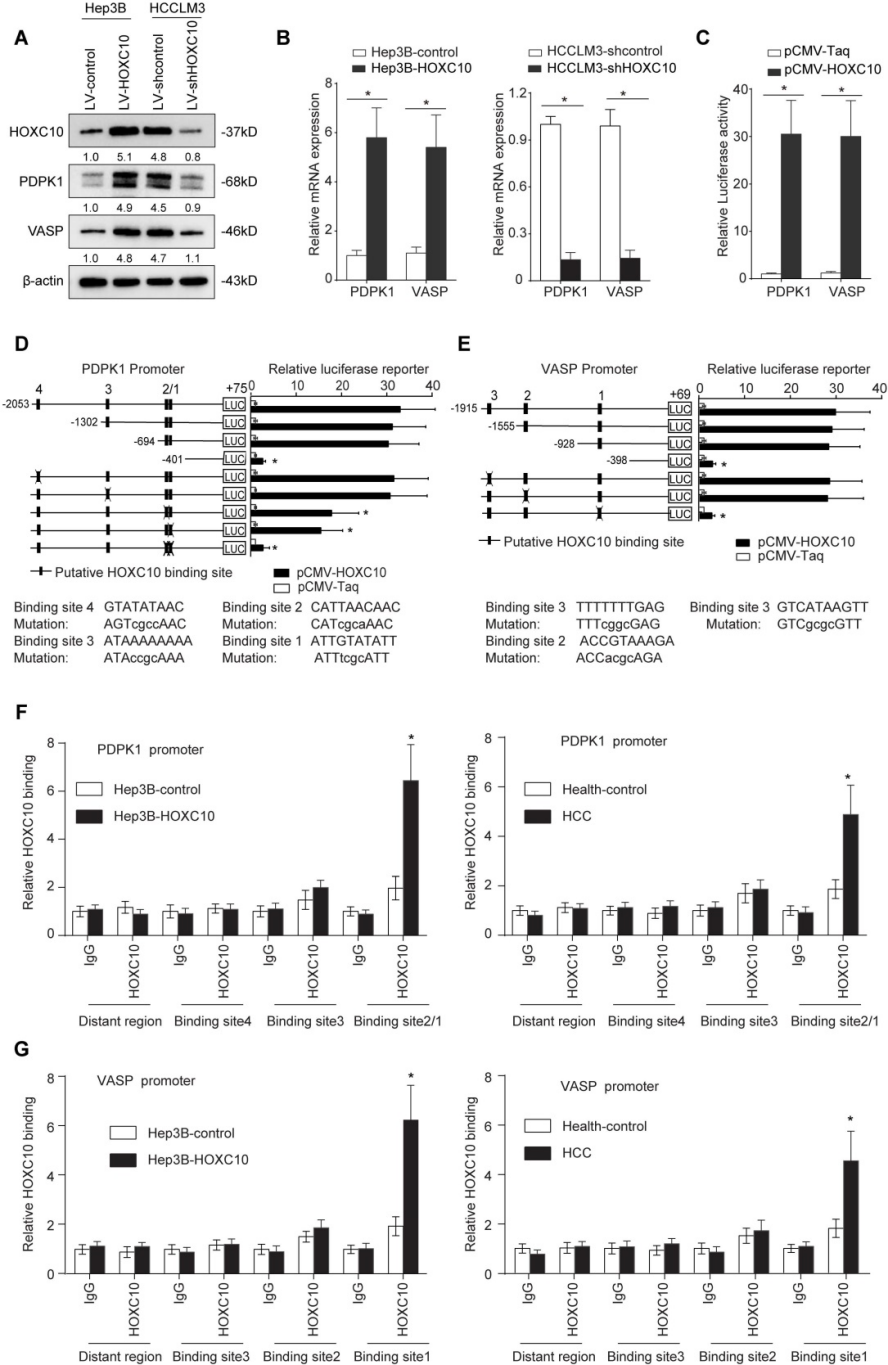
**PDPK1 and VASP are direct transcriptional targets of HOXC10.** (A) Western blotting analysis of PDPK1 and VASP expression in the indicated HCC cells. (B) Real-time PCR analysis of PDPK1 and VASP expression in the indicated HCC cells. (C) HOXC10 transactivates PDPK1 and VASP promoters. The PDPK1 or VASP promoter luciferase construct was cotransfected with pCMV-HOXC10, and promoter activities were detected using a luciferase reporter assay. (D-E) Deletion and selective mutation analyses identified HOXC10-responsive regions in the (D) PDPK1 and (E) VASP promoter. Serially truncated and mutated PDPK1 or VASP promoter constructs were cotransfected with pCMV-HOXC10, and relative luciferase activities were determined. The schematic constructs are shown (left), and the bar graphs present the relative levels of luciferase activity in each of the samples (right). (F-G) ChIP assays demonstrated the direct binding of HOXC10 to the PDPK1 (F) or VASP (G) promoter in Hep3B-HOXC10 cells (left panel) and the enriched binding of endogenous HOXC10 to the PDPK1 or VASP promoter in primary HCC tissues (right panel). Real-time PCR was performed to detect the amounts of immunoprecipitated products. Hepatocytes were separated from the liver tissues of HCC patients and healthy controls (HC). The cells were crosslinked, and the chromatin was immunoprecipitated by anti-HOXC10 or control antibody. All the data are shown as the mean±s.d. * P<0.05 ** P˂0.01.

To determine the underlying mechanism by which HOXC10 promotes HCC metastasis, we compared transcriptome changes in Hep3B-HOXC10 and Hep3B-control cells using a Affymetrix PrimeView Human Gene Expression Array. Overexpression of HOXC10 upregulated the expression of several metastasis-related genes, such as *PDPK1*, *VASP*, *BMP6* and *KRT17* (Supplementary Table S1). Considering the important role of PDPK1 20 and VASP 21 in cancer invasion and metastasis, we focused on PDPK1 and VASP for further study. Overexpression of HOXC10 significantly upregulated PDPK1 and VASP expression, whereas knockdown of HOXC10 reduced the expression levels of both genes (Figure 2A-B). The luciferase reporter assay demonstrated that the overexpression of HOXC10 promoted the luciferase activity of the *PDPK1* and *VASP* promoters (Figure 2C). We then examined the *PDPK1* and *VASP* promoter sequences and identified four and three potential HOXC10 binding motifs located in their promoters, respectively.

To identify the specific HOXC10 binding site in the *PDPK1* promoter, we generated a series of reporters containing different 5' deletions of the *PDPK1* promoter and examined their response to HOXC10 overexpression in Hep3B cells. The reporter assay showed that depletion of the cis-element located between -694 and -401 reduced the activity of the *PDPK1* promoter mediated by HOXC10 overexpression. In parallel to this result, mutation of the two putative HOXC10 binding sites in this fragment decreased HOXC10-mediated activation of the *PDPK1* promoter (Figure 2D). Likewise, mutations of the putative HOXC10 binding sites in the *VASP* promoter also reduced the HOXC10-dependent activation of the *VASP* reporter (Figure 2E). Furthermore, a chromatin immunoprecipitation assay (ChIP) demonstrated that HOXC10 binding was indeed enriched in these regions in both HCC cell lines and human HCC tissues (Figure 2F-G). Collectively, these findings indicated that PDPK1 and VASP were direct transcriptional targets of HOXC10.

### HOXC10 promotes HCC metastasis by upregulating PDPK1 and VASP expression

To study the function of PDPK1 and VASP in HCC migration and invasion, we downregulated PDPK1 and VASP expression in HCCLM3 cells, and we ectopically upregulated PDPK1 and VASP expression in Hep3B cells through lentivirus transduction (Supplementary Figure S6A). Transwell assays showed that knockdown of PDPK1 or VASP decreased the migration and invasion abilities of HCCLM3 cells (Supplementary Figure S6B-C), whereas ectopic overexpression of PDPK1 or VASP increased the migration and invasion abilities of Hep3B cells (Supplementary Figure S6D).

**Figure 3:**
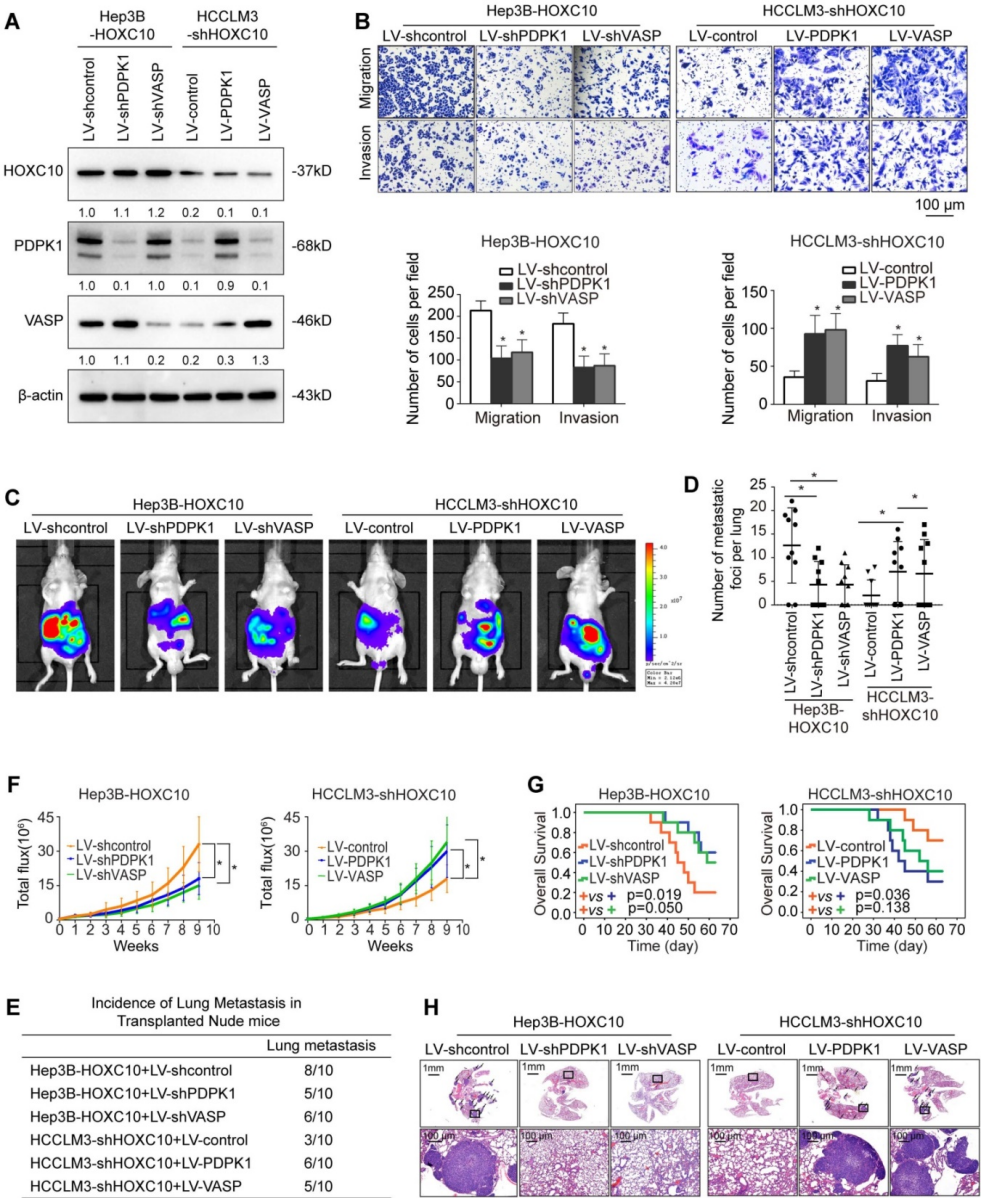
** HOXC10 promotes HCC invasion and metastasis by upregulating PDPK1 and VASP.** (A) Western blot analysis showing PDPK1 and VASP expression in Hep3B and HCCLM3 cells after lentiviral transfection. (B) Transwell assays indicated that depletion of PDPK1 and VASP inhibits the migration and invasion potentials of Hep3B-HOXC10 cells, and upregulation of PDPK1 and VASP promotes the migration and invasion abilities of HCCLM3-shHOXC10 cells. (C) The nude mice were divided into 4 groups (n=10 mice per group) and implanted with the indicated cells. Representative BLI of the different groups is shown at 9 weeks following orthotopic implantation. (D) The number of lung metastatic foci in the lung was calculated. (E) Incidence of lung metastasis in the transplanted nude mice. (F) The bioluminescent signals were recorded for 9 consecutive weeks after cell implantation. (G) The overall survival times in each group are shown. (H) Representative HE staining of lung tissues from the different groups is shown. The scale bars represent 1 mm (low magnification) and 100 μm (high magnification). All the data are shown as the mean±s.d. * P<0.05 ** P˂0.01.

To investigate whether PDPK1 and VASP are involved in HOXC10-mediated HCC metastasis, we knocked down PDPK1 and VASP expression in HOXC10-overexpressing Hep3B cells (Hep3B-HOXC10) and ectopically overexpressed PDPK1 and VASP in HCCLM3 cells with HOXC10 knockdown (HCCLM3-shHOXC10) (Figure 3A). Transwell assays showed that knockdown of PDPK1 and VASP significantly suppressed HOXC10-mediated migration and invasion capacities (Figure 3B, left and Supplementary Figure 7B upper and Supplementary Figure 8 upper) and that overexpression of PDPK1 and VASP rescued the reduced migration and invasion abilities of HCCLM3 cells with HOXC10 knockdown (Figure 3B, right). An* in vivo* metastasis assay showed that knockdown of PDPK1 and VASP reduced the incidence of lung metastasis and the number of metastatic nodules, as well as prolonged the overall survival of the Hep3B-HOXC10 group (Figure 3C-H, left and Supplementary Figure 7B lower and Supplementary Figure 8 lower). In contrast, upregulation of PDPK1 and VASP reversed the suppression of HCC metastasis in the HCCLM3-shHOXC10 group (Figure 3C-H, right). These studies suggest that HOXC10 promoted HCC metastasis by upregulating PDPK1 and VASP expression.

### HOXC10 expression is positively correlated with PDPK1 and VASP expression in human HCC tissues

**Figure 4:**
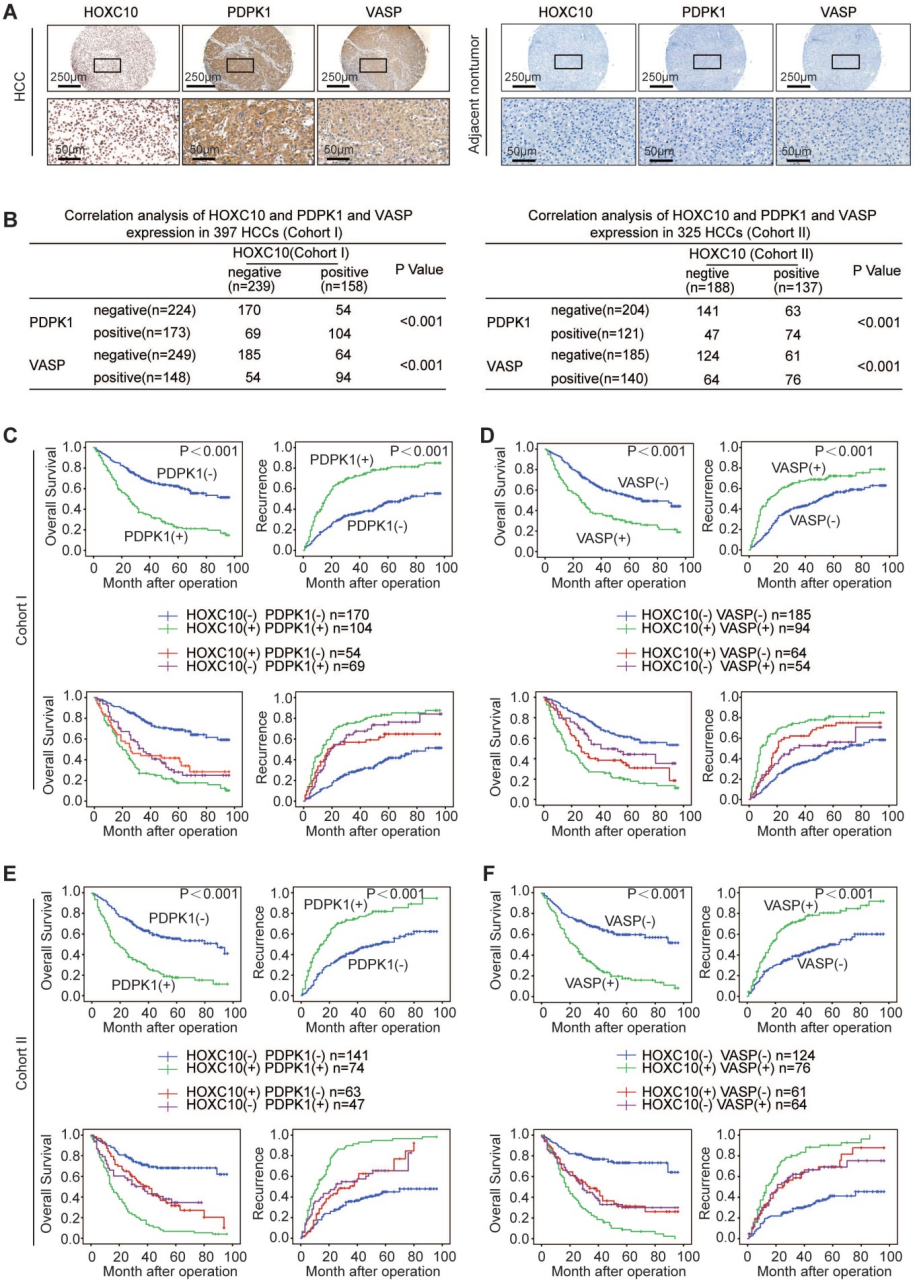
** HOXC10 expression is positively correlated with PDPK1 and VASP expression in human HCC.** (A) Representative IHC images of HOXC10, PDPK1 and VASP expression in HCC tissues and adjacent nontumorous tissues. The scale bars represent 250 µm (low magnification) and 50 µm (high magnification). (B) The correlation between the expression of HOXC10 and PDPK1 or VASP in human HCC tissues from two independent cohorts of patients. (C-D) Kaplan-Meier analysis of the correlation of PDPK1, VASP, HOXC10/PDPK1 coexpression or HOXC10/VASP coexpression with recurrence and overall survival in Cohort I. (E-F) Kaplan-Meier analysis of the correlation of PDPK1, VASP, HOXC10/PDPK1 coexpression or HOXC10/VASP coexpression with recurrence and overall survival in Cohort II.

We further evaluated the possible association between HOXC10 and PDPK1 or VASP in human HCC tissues from two independent cohorts of patients. Representative images of immunohistochemical staining of HOXC10 and PDPK1 or VASP are shown in Figure 4A. In both cohorts, HOXC10 expression was positively correlated with PDPK1 and VASP expression (Figure 4B). The overexpression of both PDPK1 and VASP was positively correlated with maximal tumor size and higher TNM stage (Supplementary Table S2-3). In addition, patients with elevated expression of PDPK1 or VASP exhibited a higher recurrence rate and poorer overall survival than patients with negative expression of PDPK1 or VASP (Figure 4C-F, upper panel). Furthermore, Kaplan-Meier analysis showed that patients with positive coexpression of either HOXC10/PDPK1 or HOXC10/VASP had the highest recurrence risk and lowest survival times in both HCC cohorts (Figure 4C-F, lower panel).

### IL-1β upregulates HOXC10 expression through the JNK/c-Jun signaling pathway

The regulatory mechanism of HOXC10 overexpression in human HCC remains unknown. Proinflammatory cytokines produced in the tumor microenvironment, including IL-1β, IL-6 and IL-8, play critical roles in promoting HCC metastasis 22. Aberrant activation of oncogenes by these proinflammatory cytokines has been reported in a wide range of HCC patients 18, 23. Considering the important roles of both proinflammatory cytokines and HOXC10 in HCC metastasis, this finding raised the question of whether proinflammatory cytokines regulate HOXC10 expression.

**Figure 5:**
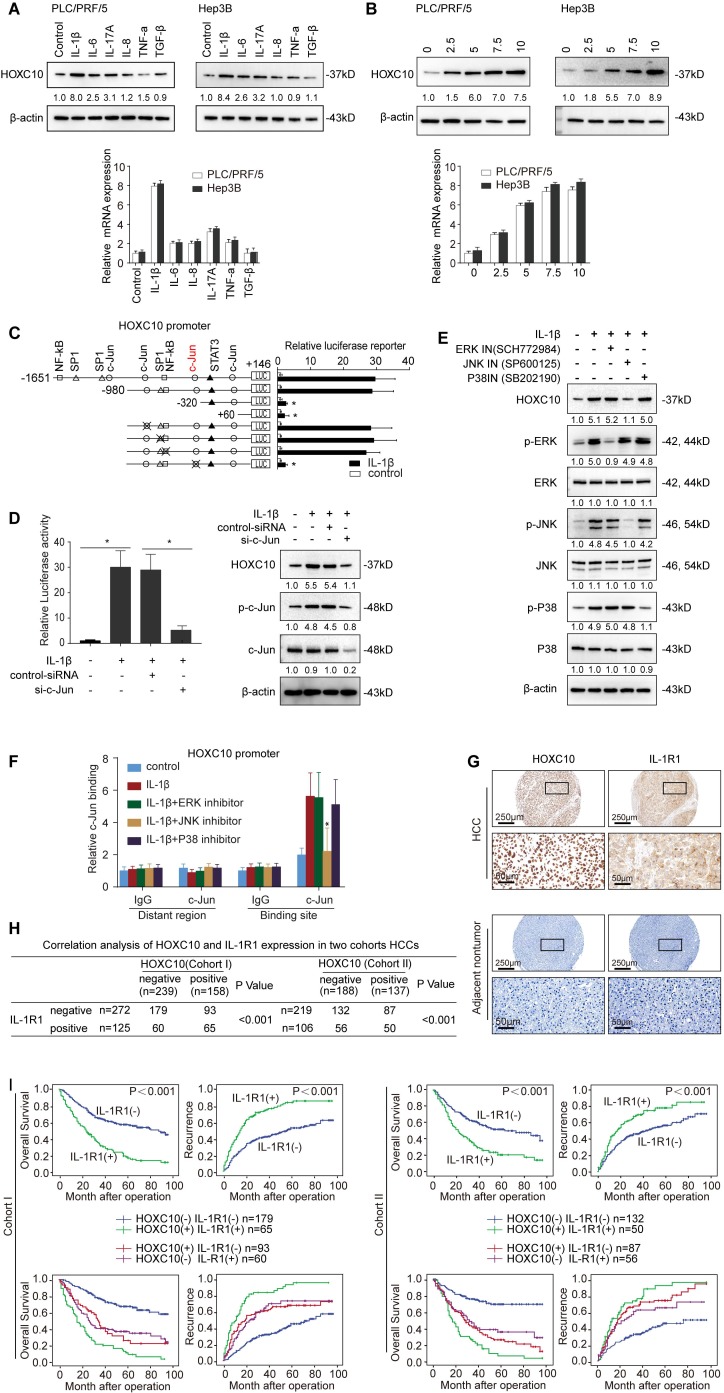
** IL-1β upregulates HOXC10 expression through the JNK/c-Jun pathway.** (A) After the indicated cells were treated with cytokines IL-1β (10 ng/ml), IL-6 (10 ng/ml), IL-8 (50 ng/ml), IL-17A (10 ng/ml), TNF-α (10 ng/ml), and TGF-β (10 ng/ml) for 24 hr, the mRNA and protein levels of HOXC10 were detected by real-time PCR and Western blotting. (B) HCC cells were treated with various concentrations of IL-1β for 24 hr, and HOXC10 expression was detected by real-time PCR and Western blotting. (C) Deletion and selective mutation analyses identified c-Jun-responsive regions in the *HOXC10* promoter. Hep3B cells were transfected with constructs with serially truncated and mutated *HOXC10* promoter constructs, and the cells were treated with or without IL-1β (10 ng/ml). Luciferase activity was measured 24 hr after IL-1β treatment. (D) Knockdown of c-Jun decreased IL-1β-induced HOXC10 overexpression. Hep3B cells were transfected with c-Jun siRNA or control siRNA and then treated with or without IL-1β. Twenty-four hours post-IL-1β treatment, HOXC10 promoter activity and expression were measured by luciferase reporter assay and Western blotting. (E) Hep3B cells were precultured with inhibitors specific to ERK, JNK and P38 and then treated with or without IL-1β. Western blotting was performed to analyze the protein expression of HOXC10 and phosphorylated and total JNK, ERK and P38. (F) A ChIP assay showed the direct binding of c-Jun to the *HOXC10* promoter induced by IL-1β, and JNK inhibitor reduced the binding of c-Jun to the *HOXC10* promoter. (G) The correlation between the expression of HOXC10 and IL-1R1 in human HCC tissues from two independent cohorts of patients. (H) Kaplan-Meier analysis of the correlation of IL-1R1 or HOXC10/IL-1R1 coexpression with recurrence and overall survival in two Cohorts.

To test this hypothesis, Hep3B and PLC/PRF/5 cells with low endogenous HOXC10 expression were treated with recombinant IL-1β, IL-6, IL-8, IL-17A, TNF-α and TGF-β. IL-1β was the most powerful inducer of HOXC10 expression in both cells (Figure 5A). To further validate these results, we treated Hep3B and PLC/PRF/5 cells with different concentrations of recombinant IL-1β (0, 2.5, 5, 7.5 and 10 ng/mL) for 24 hr. IL-1β markedly induced the expression of HOXC10 in a dose-dependent manner (Figure 5B).

To investigate the role of cis-regulatory elements of the *HOXC10* promoter in response to IL-1β stimulation, the -1651 to +146 bp region and a series of truncations and mutations of the human *HOXC10* promoter were generated. A significant reduction in IL-1β-induced *HOXC10* promoter activity was observed when Hep3B cells were transfected with the truncated (-980 ~ -320) *HOXC10* promoter, indicating that this sequence between (-980 ~ -320) was crucial for the activation of the *HOXC10* promoter induced by IL-1β. Two c-Jun binding sites, one SP1 binding site and one NF-κB binding site are located in this region. Site-directed mutagenesis showed that mutation of the c-Jun binding site (located at the -359 site) significantly reduced the *HOXC10* promoter activity induced by IL-1β, while mutation of the other c-Jun (located at the -803 site), SP1 and NF-κB binding sites had no significant effect on *HOXC10* promoter activity induced by IL-1β (Figure 5C). Knockdown of c-Jun markedly reduced the enhanced promoter activity and expression of HOXC10 induced by IL-1β treatment (Figure 5D).

IL-1β signaling has been reported to activate the ERK, P38 and JNK pathways 16. We treated the cells with inhibitors of ERK (SCH772984), P38 kinase (SB202190) and JNK (SP600125) to identify which signaling pathway was responsive to IL-1β-mediated HOXC10 expression. The protein levels of HOXC10 and of phosphorylated and total ERK, JNK and P38 were analyzed by Western blotting. Pretreatment of cells with the JNK inhibitor significantly decreased IL-1β-mediated HOXC10 expression, whereas pretreatment of cells with the ERK or P38 inhibitor showed no such effect (Figure 5E). Consistently, a ChIP assay demonstrated that the JNK inhibitor markedly inhibited the binding of c-Jun to the *HOXC10* promoter, while the ERK and P38 inhibitors showed little effect on the binding of c-Jun to the *HOXC10* promoter (Figure 5F). These data suggested that IL-1β upregulated HOXC10 expression through the JNK/c-Jun signaling pathway.

To investigate the clinical relevance of IL-1R1, which is the receptor of IL-1β, IHC analysis was performed in two independent HCC cohorts. IL-1R1 expression was significantly upregulated in HCC tissues compared with adjacent nontumor tissues, and typical IHC images are shown in Figure 5G. In both cohorts, overexpression of IL-1R1 was positively correlated with maximal tumor size and higher TNM stage (Supplementary Table S4). IL-1R1 expression was positively correlated with HOXC10 expression (Figure 5H). In addition, patients with positive expression of IL-1R1 exhibited a higher recurrence rate and poorer overall survival time than patients with negative expression of IL-1R1 (Figure 5I, upper panel). Furthermore, Kaplan-Meier analysis showed that patients with positive coexpression IL-1R1/HOXC10 had the highest recurrence risk and lowest survival times in both HCC cohorts (Figure 5I, lower panel). To further investigate the role of HOXC10 and PDPK1, VASP or IL-1β receptor IL-1R1 in human HCC metastasis, immunohistochemistry and RT-PCR was used to detect their expression in 20 paired primary and metastatic HCC tissues. A representative case of immunohistochemical staining of all three markers was shown in Supplementary Figure S3A. A higher level of HOXC10 and PDPK1, VASP or IL-1R1expression was observed in metastatic HCC tissues than in primary HCC samples and adjacent nontumor tissues (Supplementary Figure S3A-B).

### HOXC10 is essential for IL-1β-mediated HCC metastasis

**Figure 6:**
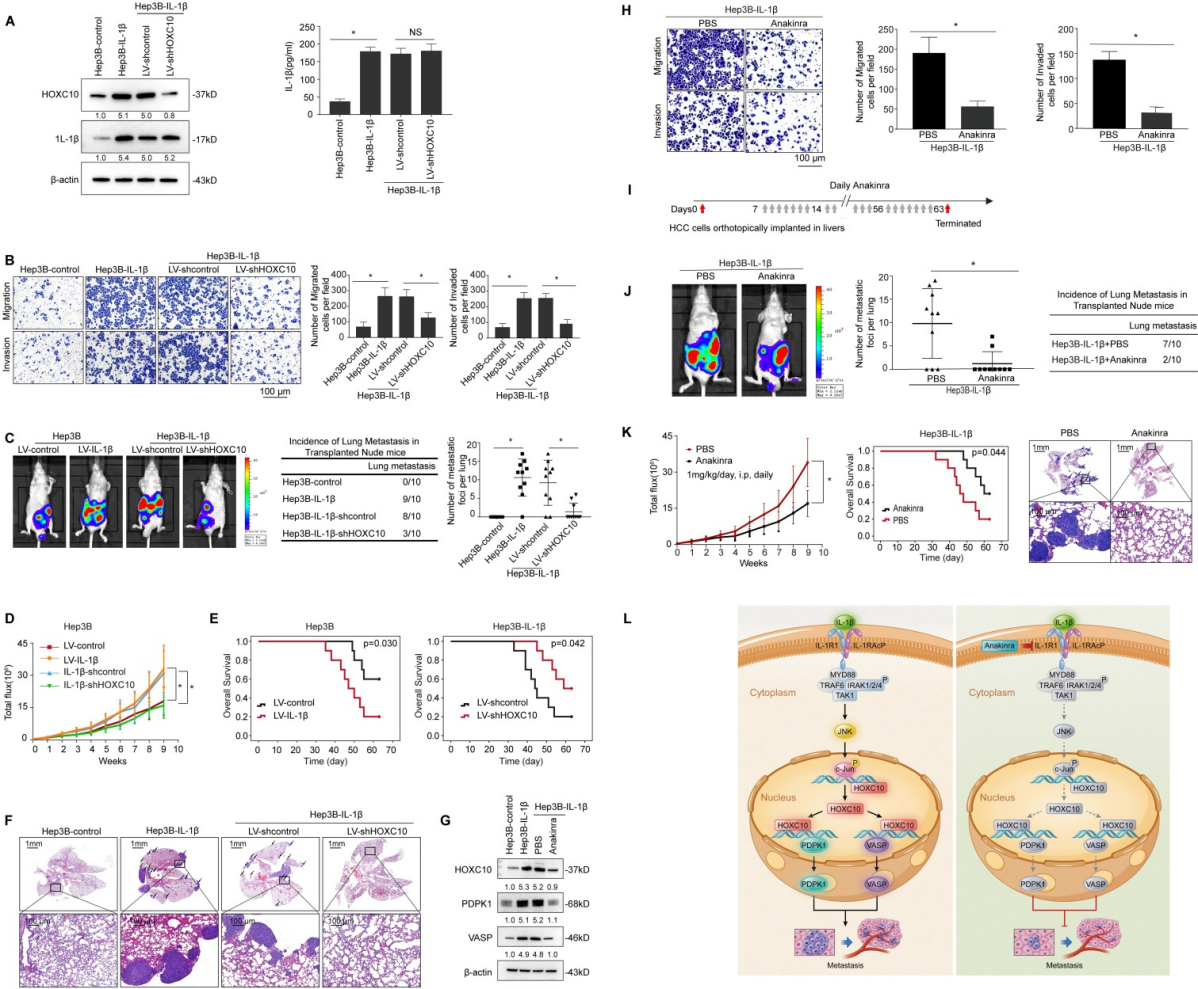
** HOXC10 is essential for IL-1β-mediated HCC metastasis expression. (A)** Hep3B-IL-1β cells were infected with LV-shcontrol or LV-shHOXC10 by lentiviral transduction, and HOXC10 expression was examined by Western blotting. The IL-1β levels in the supernatant of the indicated cells were detected by enzyme-linked immunosorbent assay (ELISA).** (B)** Transwell assays showed that HOXC10 knockdown inhibited the migration and invasion abilities of Hep3B-IL-1β cells.** (C-F)** Knockdown of HOXC10 inhibited IL-1β-mediated HCC metastasis. (C) Bioluminescence images, metastasis incidence, and number of lung metastasis foci of the indicated groups of nude mice are shown. (D) Bioluminescence signals. (E) Overall survival. (F) Representative HE staining of lung tissues from the different groups is shown. The scale bars represent 1 mm (low magnification) and 100 μm (high magnification).** (G)** After Hep3B-IL-1β cells were treated with Anakinra (10 μg/ml) for 24 hr, the protein levels of HOXC10, PDPK1 and VASP were detected by Western blotting.** (H)** Anakinra treatment (10 μg/ml, 24 hr) significantly inhibited the migration and invasion abilities of Hep3B-IL-1β cells. **(I-K)** Anakinra treatment markedly inhibited IL-1β-mediated HCC metastasis. (I) Anakinra, 1 mg/kg/day, or PBS, was administered intraperitoneally for 9 weeks. starting 1 week after orthotopic implantation of the tumor. (J) The bioluminescent signals, numbers of lung metastatic foci and incidence of lung metastasis. **(K)** The overall survival times and representative HE staining of lung tissues from the different groups are shown. The scale bars represent 1 mm (low magnification) and 100 μm (high magnification). **(L)** A schematic diagram of the role of IL-1β-HOXC10 signaling in inflammation-related HCC metastasis. IL-1β-IL-1R1 signaling upregulates HOXC10 expression through the JNK/c-Jun signaling pathway. PDPK1 and VASP are direct transcriptional targets of HOXC10. HOXC10 promotes HCC invasion and metastasis by upregulating PDPK1 and VASP expression. The IL-1R1 antagonist Anakinra inhibits IL-1β-mediated HOXC10 upregulation, thereby inhibiting IL-1β-HOXC10 signaling-mediated HCC invasion and metastasis.

As HOXC10 was upregulated by IL-1β and contributed to HCC metastasis, we subsequently investigated its function in IL-1β-mediated HCC metastasis. We downregulated HOXC10 expression via lentiviral transduction in IL-1β-overexpressing cells (Hep3B-IL-1β), and the expression level of HOXC10 was evaluated by Western blotting (Figure 6A). Upregulation of IL-1β increased the migration and invasion abilities of Hep3B cells, and knockdown of HOXC10 significantly reduced IL-β-mediated migration and invasion abilities (Figure 6B). In addition, treatment of Hep3B cells with recombinant IL-1β significantly increased migration and invasion abilities of Hep3B cells, and knockdown of HOXC10 dramatically decreased cell migration and invasion abilities induced by recombinant IL-1β stimulation (Supplementary Figure S4). In an* in vivo* metastasis assay, overexpression of IL-1β increased the incidence of lung metastasis and the number of metastatic lung nodules and decreased the overall survival in Hep3B cells compared with that in control cells. However, downregulation of HOXC10 decreased the incidence of lung metastasis and the number of metastatic lung nodules while increasing the overall survival in the Hep3B-IL-1β xenograft group (Figure 6C-F and Supplementary Figure S6C).

In addition, since HCCLM3 cells (with high metastatic capacity) have highly endogenous IL1β expression 18, we downregulated IL-1β expression through lentiviral transduction in HCCLM3 cells and ectopically overexpressed HOXC10 expression in HCCLM3-shIL-1β cells (Supplementary Figure S9A). Transwell assays showed that downregulation of IL-1β inhibited the migration and invasion abilities of HCCLM3 cells, whereas ectopic overexpression of HOXC10 rescued the reduced migration and invasion abilities induced by IL-1β knockdown (Supplementary Figure S9B). An *in vivo* metastasis assay showed that knockdown of IL-1β reduced the incidence of lung metastasis and the number of metastatic nodules, as well as prolonged the overall survival of the HCCLM3 cells group. In contrast, the upregulation of HOXC10 rescued the decreased incidence of lung metastasis and the number of metastatic lung nodules while increasing the overall survival time of the HCCLM3-shIL-1β group (Supplementary Figure S9C-G). These results suggest that HOXC10 is critical for IL-1β-enhanced HCC invasion and metastasis.

IL-1R1 antagonist (IL-1Ra) Anakinra has been approved by the FDA for the treatment of RA 24. Previous studies reported that Anakinra treatment reduced pancreatic cancer cell migration and invasion 25 and inhibited tumor growth 26 and metastasis 27 in a murine breast cancer model. We aimed to determine whether Anakinra treatment affects IL-1β-HOXC10 signaling-mediated HCC invasion and metastasis. Western blotting analysis showed that Anakinra treatment significantly suppressed the expression of HOXC10 and its target genes PDPK1 and VASP in Hep3B-IL-1β cells (Figure 6G). Anakinra treatment suppressed the migration and invasion abilities of Hep3B-IL-1β cells (Figure 6H). Daily administration of Anakinra significantly reduced lung metastasis and prolonged survival time of the Hep3B-IL-1β group compared to that of the control group (Figure 6I-K and Supplementary Figure S6B lower). These data indicated that the IL-1R1 antagonist Anakinra inhibited IL-1β-mediated HOXC10 upregulation and HCC invasion and metastasis.

## Discussion

Metastasis is the major reason for high recurrence rates and poor survival times among patients with HCC 28. In this study, we found that HOXC10 expression was markedly higher in HCC tissues than in adjacent noncancerous tissues. Overexpression of HOXC10 positively correlated with maximal tumor size, tumor encapsulation loss and high TNM stage and was an independent risk factor for higher recurrence and shorter overall survival in HCC patients. In addition, HOXC10 expression was much higher in HCC tissues from patients who developed metastasis than in HCC tissues from patients who did not develop metastasis. Furthermore, our *in vitro* and *in vivo* studies showed that upregulation of HOXC10 promoted HCC invasion and metastasis and that downregulation of HOXC10 inhibited HCC invasion and metastasis. Taken together, both the clinical data and the experimental results suggest that HOXC10 plays an important role in promoting HCC metastasis.

Previous studies reported that PDPK1 was significantly upregulated in several human cancers, such as melanoma, multiple myeloma, head and neck carcinoma, and HCC 29-32. Overexpression of PDPK1 promoted cancer migration, invasion, and distant metastasis by regulating AKT phosphorylation 33-34. VASP, a member of the Ena/VASP family, regulates actin cytoskeleton and cell migration 35. Overexpression of VASP promotes cancer cell invasion and metastasis and is positively associated with poor TNM stage and poor prognosis in several human cancers, including HCC 21, 36-38. These studies suggest that both PDPK1 and VASP play important roles in cancer invasion and metastasis. In this study, we found that PDPK1 and VASP are transcriptional targets of HOXC10. Knockdown of PDPK1 and VASP dramatically inhibited HOXC10-mediated HCC metastasis, whereas ectopic overexpression of PDPK1 and VASP rescued the HOXC10 knockdown-mediated decrease in HCC metastasis. In human HCC tissues from two independent cohorts of patients, HOXC10 expression was positively correlated with PDPK1 and VASP expression, and patients with positive coexpression of HOXC10/PDPK1 or HOXC10/VASP showed the poorest overall survival and highest recurrence rate. These data indicated that HOXC10 promoted HCC metastasis by upregulating PDPK1 and VASP expression.

The IL-1 pathway is triggered when the ligands IL-1α and IL-1β bind to IL-1R1, which forms a complex with the IL-1 receptor accessory protein (IL-1RAcP) 16. Anakinra, a specific IL-1R antagonist, has been approved by the FDA for the treatment of RA 26. Recent studies have reported that the IL-1β/IL-1R1 signaling pathway promotes cancer proliferation, invasion and metastasis in several cancers. Inhibition of IL-1β/IL-1R1 signaling by Anakinra treatment significantly inhibited cancer proliferation and metastasis 27-29. In this study, we found that IL-1R1 expression was significantly upregulated in human HCC tissues. Overexpression of IL-1R1 was positively correlated with the loss of tumor encapsulation and with microvascular invasion and higher TNM stage. HCC patients with positive IL-1R1 expression had shorter overall survival and higher recurrence rates than those with negative IL-1R1 expression. In addition, HOXC10 expression positively correlated IL-1R1 expression. Patients with positive co-expression of IL-1R1 and HOXC10 had the poorest overall survival and highest recurrence rates. Furthermore, Anakinra suppressed IL-1β-mediated HOXC10 upregulation and HCC invasion and metastasis *in vitro* and *in vivo*. These studies suggested that targeting the IL-1β/IL-1R1/HOXC10 pathway may provide a promising strategy for the inhibition of HCC metastasis.

In summary, we reported a role for HOXC10 in HCC metastasis. HOXC10 promoted HCC metastasis by upregulating PDPK1 and VASP expression. IL-1β/IL-1R1 signaling upregulated HOXC10 expression via the JNK/c-Jun pathway. Anakinra, a specific antagonist for IL-1R1, inhibited IL-1β-induced HOXC10 upregulation and HCC metastasis. Thus, HOXC10 is a prognostic biomarker in human HCC, and targeting this signaling pathway may provide evidence for the development of potential treatment strategies for HCC.

## Supplementary Material

Supplementary figures and tables.

## Figures and Tables

**Table 1:** Correlation between HOXC10 expression and clinicopathological characteristics of HCCs in two independent cohorts of human HCC tissues

Clinicopathological variables	Cohort I				Cohort II		
	Tumor HOXC10 expression			*P* Value	Tumor HOXC10 expression		*P* Value
	Negative (n=239)		Positive (n=158)		Negative (n=188)	Positive (n=137)	
Age ≤50		91	67	0.388	39	25	0.576
>50		148	91		149	112	
Sex	female	56	29	0.227	31	16	0.223
	male	183	129		157	121	
Serum AFP	≤20ng/ml	182	116	0.538	57	40	0.827
	>20ng/ml	57	42		131	97	
Virus infection	HBV	172	110	0.741	131	101	0.761
	HCV	21	13		16	8	
	HBV+HCV	13	7		12	7	
	None	33	28		29	21	
Cirrrhosis	absent	64	53	0.148	46	32	0.817
	present	175	105		142	105	
Child-pugh score	Class A	184	122	0.958	153	109	0.682
	Class B	55	36		35	28	
Tumor number	single	199	115	0.01	139	89	0.081
	multiple	40	43		49	48	
Maximal tumor size	≤5cm	151	62	<0.001*	97	48	0.003
	>5cm	88	96		91	89	
Tumor encapsulation	absent	110	51	0.006	60	64	0.007
	present	129	107		128	73	
Microvascular invasion	absent	138	67	0.003	115	79	0.525
	present	101	91		73	58	
Tumor differentiation	I-II	157	108	0.581	137	91	0.210
	III-IV	82	50		51	46	
TNM stage	I-II	173	77	<0.001*	142	84	0.006
	III	66	81		46	53	

**Table 2:** Univariate and multivariate analysis of factors associated with survival and recurrence in two independent cohorts of human HCC.

Clinical Variables	Time To Recurrence		Overall Survival	
	HR( 95% CI )	P value	HR(95% CI)	P value
**Cohort I(n=397)**				
**Univariate analysis**				
Age (≤50 versus > 50)	0.914 (0.702-1.190)	0.504	0.941 (0.717-1.234)	0.659
Sex (female versus male)	1.101 (0.812-1.492)	0.536	1.131 (0.826-1.547)	0.442
Serum AFP (≤20 versus >20 ng/ml)	1.230 (0.909-1.665)	0.179	1.272 (0.931-1.737)	0.131
HBV infection (no versus yes)	1.097 (0.814-1.479)	0.543	1.211(0.892-1.645)	0.220
Cirrhosis ( absent versus present)	1.101 (0.830-1.460)	0.505	1.270 (.952-1.694)0	0.104
Child-pugh score (A versus B)	0.964 (0.716-1.296)	0.806	1.206 (0.873-1.665)	0.256
Tumor number (single versus multiple)	0.632 (0.470-0.851)	0.003	0.628 (0.465-0.849)	0.003
Maximal tumor size (≤5cm versus >5)	0.234 (0.176-0.309)	<0.001	0.174 (0.129-0.235)	<0.001
tumor encapsulation (present versus absent)	0.305 (0.225-0.414)	<0.001	0.231(0.166-0.322)	<0.001
Microvascular invasion (absent versus present)	0.325 (0.248-0.425)	<0.001	0.241(0.180-0.321)	<0.001
Tumor differentiation (I-II versus III-Ⅳ)	0.804 (0.706-0.916)	0.001	0.920 (0.801-1.056)	0.237
TNM stage (I-II versus III)	0.187 (0.142-0.247)	<0.001	0.132 (0.098-0.177)	<0.001
HOXC10 expression (negative versus positive)	0.414 (0.320-0.536)	<0.001	0.359 (0.275-0.467)	<0.001
**Multivariate analysis**				
Tumor number (single versus multiple)	0.705 (0.521-0.954)	0.024	0.681 (0.501-0.926)	0.014
Maximal tumor size (≤5cm versus >5)	0.507 (0.357-0.722)	<0.001	0.419 (0.288-0.611)	0.190
Tumor encapsulation (present versus absent)	0.579 (0.413-0.812)	0.002	0.503 (0.350-0.724)	<0.001
Microvascular invasion (absent versus present)	0.806 (0.575-1.131)	0.212	0.636 (0.447-0.940)	0.012
Tumor differentiation (I-II versus III-Ⅳ)	0.645 (0.496-0.840)	0.001	0.829 (0.629-1.097)	0.190
TNM stage (I-II versus III)	0.316 (0.277-0.439)	<0.001	0.220 (0.155-0.311)	<0.001
HOXC10 expression (negative versus positive)	0.468 (0.358-0.699)	<0.001	0.413 (0.312-0.547)	<0.001
				
**Cohort II(n=325)**				
**Univariate analysis**				
Age (≤50 versus > 50)	0.918 (0.641-1.313)	0.638	0.770 (0.527-1.123)	0.174
Sex (female versus male)	0.754 (0.499-1.140)	0.181	0.643 (0.408-1.012)	0.058
Serum AFP (≤20 versus >20 ng/ml)	0.876 (0.643-1.192)	0.398	0.796 (0.578-1.098)	0.164
HBV infection (no versus yes)	1.004 (0.737-1.366)	0.981	0.947 (0.686-1.308)	0.742
Cirrhosis (absent versus present)	1.019 (0.733-1.418)	0.910	0.983 (0.699-1.383)	0.924
Child-pugh score (A versus B)	0.898 (0.628-1.278)	0.393	0.855 (0.596-1.225)	0.393
Tumor number (single versus multiple)	0.537 (0.428-0.767)	<0.001	0.523 (0.389-0.703)	<0.001
Maximal tumor size (≤5cm versus >5)	0.506 (0.378-0.677)	<0.001	0.446 (0.328-0.607)	<0.001
Tumor encapsulation (present versus absent)	0.537 (0.405-0.713)	<0.001	0.469 (0.350-0.627)	<0.001
Microvascular invasion (absent versus present)	0.561 (0.423-0.743)	<0.001	0.557 (0.433-0.770)	<0.001
Tumor differentiation (I-II versus III-Ⅳ)	0.638 (0.509-0.916)	0.011	0.178 (0.533-0.969)	0.030
TNM stage (I-II versus III)	0.391 (0.290-0.521)	<0.001	0.292 (0.217-0.393)	<0.001
HOXC10 expression (negative versus positive)	0.338 (0.253-0.451)	<0.001	0.347 (0.258-0.467)	<0.001
**Multivariate analysis**				
Tumor number (single versus multiple)	0.705 (0.521-0.954)	0.024	0.618 (0.501-0.926)	0.014
Maximal tumor size (≤5cm versus >5)	0.507 (0.357-0.722)	<0.001	0.419 (0.288-0.611)	0.190
Tumor encapsulation (present versus absent)	0.579 (0.413-0.812)	0.002	0.503 (0.350-0.724)	<0.001
Microvascular invasion (absent versus present)	0.806 (0.575-1.131)	0.212	0.636 (0.447-0.940)	0.012
Tumor differentiation (I-II versus III-Ⅳ)	0.645 (0.496-0.840)	0.001	0.829 (0.629-1.097)	0.190
TNM stage (I-II versus III)	0.316 (0.277-0.439)	<0.001	0.220 (0.155-0.311)	<0.001
HOXC10 expression (negative versus positive)	0.468 (0.358-0.699)	<0.001	0.413 (0.312-0.547)	<0.001

## Abbreviations

HCC: hepatocellular carcinoma
TNM: tumor-node-metastasis
HE: hematoxylin and eosin
IHC: immunohistochemistry
mRNA: messenger RNA
shRNA: short hairpin RNA
ChIP: chromatin immunoprecipitation
BLI: bioluminescent imaging
TSG: tumor suppressor gene
HOXC10: homeobox C10
PDPK1: phosphoinositide dependent protein kinase 1
VASP: vasodilator-stimulated phosphoprotein
JNK: c-Jun NH2-terminal kinase
