# Supplementary material for: Interleukin 1β-mediated HOXC10 Overexpression Promotes Hepatocellular Carcinoma Metastasis by Upregulating PDPK1 and VASP
Source: Theranostics. 2020 Feb 19;10(8):3833–48. doi: 10.7150/thno.41712 (PMC7069084; doi:10.7150/thno.41712)
Supplement: Supplementary file 1 — Supplementary figures and tables. [file thnov10p3833s1.pdf]

Supplementary Material and Methods

Cell culture

Immortalized liver cell lines (HL7702) and HCC cells (SNU878) were purchased from the Institute of Biochemistry and Cell Biology, Chinese Academy of Science, China. Human HCC cells (HepG2, Huh-7, Hep3B, PLC/PRF/5, SNU387, SNU398 and SNU449) were purchased from the American Type Culture Collection. Additional human HCC cells (MHCC97H, HCCLM3, and HCCLM6) were kindly provided by Dr. Tang ZY (Liver Cancer Institute, Zhongshan Hospital, Fudan University, Shanghai, China). MHCC97H, HCCLM3, and HCCLM6 cells are stepwise metastatic potential cell lines with the same genetic background but different lung metastatic potentials. HepG2, Hep3B, Huh7, and PLC/PRF/5 are HCC cells with low metastatic potential, whereas MHCC97H, HCCLM3, and HCCLM6 are HCC cells with high metastatic potential. All cell lines were cultured in Dulbecco's modified Eagle's medium (DMEM) supplemented with 10% FBS, 100 μg/ml streptomycin and 100 μg/ml penicillin.

Plasmid construction

Plasmid construction was performed according to standard procedures as outlined in our previous study[1]. The primers are presented in Supplementary Table S5. For example, the PDPK1 promoter construct, (-2053/+75) PDPK1, was generated from human genomic DNA. This construct corresponds to the sequence from -2053 to +75 (relative to the transcriptional start site) of the 5'-flanking region of the human

PDPK1 gene. It was generated with forward and reverse primers incorporating KpnI and XhoI sites at the 5’ and 3’-ends, respectively. The polymerase chain reaction (PCR) product was cloned into the KpnI and XhoI sites of the pGL3-Basic vector (Promega). The 5’-flanking deletion constructs of the PDPK1 promoter, (-1302/+75) PDPK1, (-694/+75) PDPK1, (-401/+75) PDPK1 were similarly generated using the (-2053/+75) PDPK1 construct as the template. The HOXC10 binding sites in the PDPK1 promoter were mutated using the QuikChange II Site-Directed Mutagenesis Kit (Stratagene). The constructs were confirmed by DNA sequencing. Other promoter constructs were cloned in the same manner.

Construction of lentivirus and stable cell lines

Lentiviral vectors encoding shRNAs were generated using PLKO.1-TRC (Addgene) and designated as LV-shHOXC10, LV-shPDPK1, LV-shVASP, LV-shHOXA3, LV-shHOXA6, LV-shHOXA10, LV-shHOXA13, LV-shHOXB5, LV-shHOXB7, LV-shHOXC4，LV-shHOXD9 and LV-shcontrol. “LV-shcontrol” is a non-target shRNA control. The vector “PLKO.1-puro Non-Target shRNA Control Plasmid DNA” (purchased from Sigma, SHC016) contains an shRNA insert that does not target any known genes from any species. The shRNA sequences can be found in Supplementary Table S6. Lentiviral vectors encoding the human HOXC10，PDPK1 and VASP genes were constructed in FUW-teto (Addgene) and designated as LV-HOXC10, LV-PDPK1 and LV-VASP. An empty vector was used as the negative control and was designated as LV-control.

Concentrated lentivirus was transfected into the HCC cells with a multiplicity of infection (MOI) ranging from 20 to 50 in the presence of polybrene (5 μg/ml). Seventy-two hours after infection, HCC cells were selected for 2 weeks using 2.5 μg/ml puromycin (OriGene). Selected pools of knockdown and over-expressing cells were used for the following experiments.

In vitro invasion and migration assay

For the migration and invasion assay, a 24 well chamber with 8-μm pore filter (Corning corporation, USA) was used. For migration assay, 5×10^5 cells were seeded into the upper chamber in serum-free medium. For invasion assay, 5×10^5 cell were implanted in the top chamber with Matrigel (Corning corporation, USA). After 24-48 hours, the cells were fixed with 95% ethanol and stained with crystal violet. The mean of triplicate assays for each experimental condition was used.

Animal experiment

All animal studies were approved by the Committee on the Use of Live Animals in Teaching and Research, Fourth Military Medical University. Five-weeks-old BALB/C male nude mice were raised in specific pathogen-free conditions in accord with the institutional guidelines for animal care. For *in vivo* metastasis assay, human luciferase labeled HCC cells (4.0×10^6) in the 100 μl of phosphate-buffered saline that were mixed with 100 μl matrigel were injected into the right lobes of livers of the nude mice under anesthesia (10 for each group). The *in vivo* tumor formation and

metastases were monitored using the bioluminescence. For *in vivo* signal detection, D-luciferin (Perkin-Elmer) at 100 mg/kg was injected intraperitonially into the nude mice. Bioluminescent images were captured using an IVIS 100 Imaging System (Xenogeny). At the 9 weeks, the mice were sacrificed and the livers and lungs were collected and underwent histological examination.

Western Blotting

For Western blotting assay, the lysed cells protein was separated on SDS-PAGE and transferred onto polyvinylidene difluoride membrane. The nonspecific binding was blocked with 10% non-fat milk for one hour. The membranes were incubated with specific antibody overnight at 4°C. Western blotting of β-actin on the same membrane was used as a loading control. Antibody against HOXC10 (11230-3F2) was purchased from abnova. Antibody against β-actin (A1978) was purchased from sigma. Antibodies against VASP (ab229624) and IL-1R1(ab154524a), HOXA3(ab28771), HOXA6(ab74064), HOXA10(ab191470), HOXA13(ab106503), HOXB5(ab229345), HOXB7(ab152454), HOXC4(ab230629) and HOXD9(ab90260) were purchased from abcam. Antibodies against p-Akt (Ser308, #4060), Akt (#4691), Cleaved IL-1β (#83186), p38 MAPK (#8690), p-p38MAPK (#4511), Erk1/2 (#4695), p-Erk1/2 (#4730), c-Jun (#9165), p-c-Jun (#9255), JNK (#9252) and p-JNK (#4668) was purchased from Cell Signaling. Antibody against PDPK1(sc-7765) was purchased from Santa Cruz Biotechnology. The membranes were then washed with PBS 3 times and incubated with an HRP-conjugated secondary antibody. Proteins were visualized

using a Immobilon^{TM} Western Chemiluminescent HRP substrate (Millipore, USA).

Real-time PCR

Total RNA was extracted using TRIzol Reagent (Invitrogen), and reverse transcription was performed using the Advantage RT-for-PCR Kit (Takara) according to the manufacturer's instructions. For the real-time PCR analysis, aliquots of double-stranded cDNA were amplified using a SYBR Green PCR Kit (Applied Biosystems). The cycling parameters were as follows: 95 ℃ for 15 s, 55-60 ℃ for 15 s, and 72 ℃ for 15 s for 45 cycles. A melting curve analysis was then performed. The Ct was measured during the exponential amplification phase, and the amplification plots were analyzed using SDS 1.9.1 software (Applied Biosystems). For the cell lines, the relative expression levels (defined as the fold change) of the target genes were determined by the following equation: $2^{-\Delta\Delta Ct}$ ($\Delta Ct = \Delta Ct^{target} - \Delta Ct^{GAPDH}$, $\Delta\Delta Ct = \Delta Ct^{expressing\ vector} - \Delta Ct^{control\ vector}$). The expression level was normalized to the fold change that was detected in the corresponding control cells, which was defined as 1.0. For the clinical tissue samples, the fold change of the target gene was determined by the following equation: $2^{-\Delta\Delta Ct}$ ($\Delta\Delta Ct = \Delta Ct^{tumor} - \Delta Ct^{nontumor}$). This value was normalized to the average fold change in the normal liver tissues, which was defined as 1.0. All reactions were performed in duplicate. The primer sequences are listed in Supplementary Table S5.

Luciferase reporter assays

To examine the signaling pathways regulated by HOXC10 and the downstream modulated by HOXC10, the luciferase activity was performed by the Dual Luciferase Assay (Promega, Madison, WI), following to the manufacturer's instructions. Then, the transfected cell was lysed in culture dishes which containing a lysis buffer, and the resulting lysates were centrifuged for 1 minute at maximum speed in an Eppendorf microcentrifuge. Relative luciferase activity (RLA) was analyzed by TD20/20 Luminometer (Turner Biosystems, Sunnyvale, CA), and the transfection efficiencies were normalized according to Rinella activity.

Reagent

ERK inhibitor SCH772984 (#S7101), p38 inhibitor SB202190(#S1077) and JNK inhibitor SP600125 (#S1460) were purchased from Selleck (Houston, TX, USA). Anakinra were purchased from (Amgen, Cambridge, UK). All the reagents were used according to the manufacturer's instruction.

Reference

1. Xia L, Huang W, Tian D, Zhu H, Zhang Y, Hu H, Fan D, et al. Upregulated FoxM1 expression induced by hepatitis B virus X protein promotes tumor metastasis and indicates poor prognosis in hepatitis B virus-related hepatocellular carcinoma. J Hepatol 2012; 57: 600-612.

Supplementary Figure S1

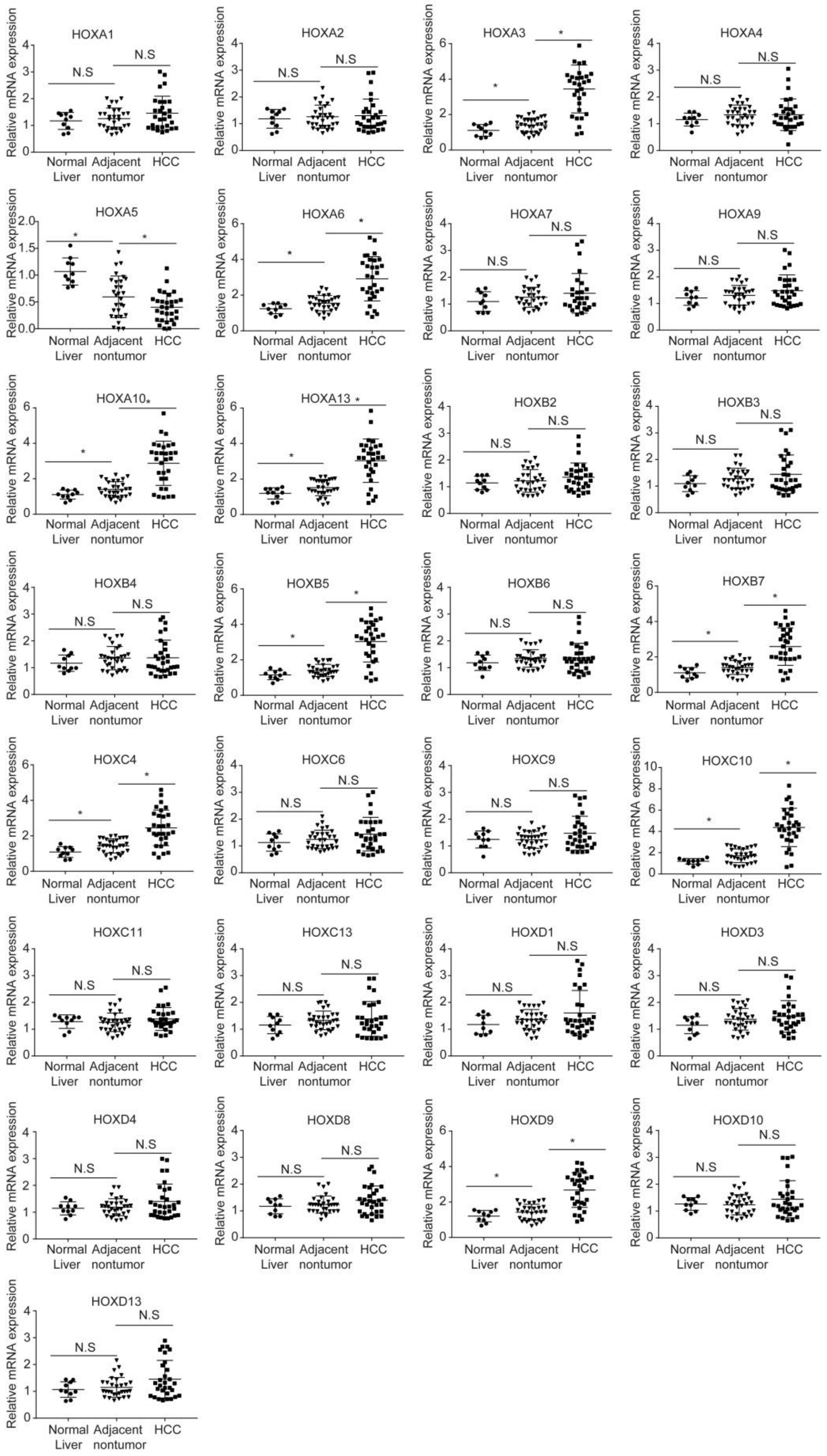

Supplementary Figure S1. Relative mRNA expression of HOX family members in normal liver tissues (n=10) and 30 paired of HCC tissues and adjacent nontumor tissues.

Supplementary Figure S2

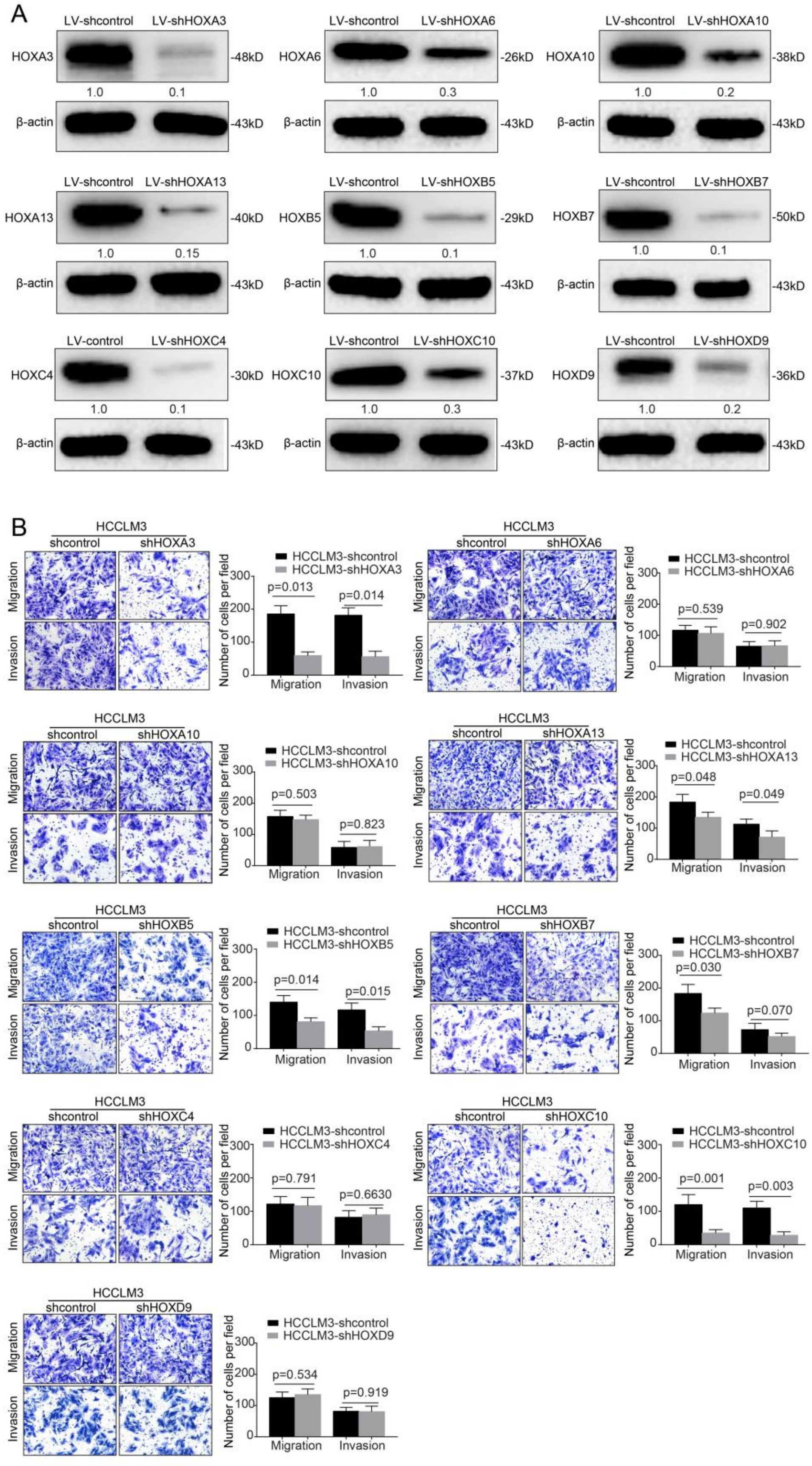

Supplementary Figure S2. The effect of the selected 9 HOX genes on the proliferation, invasion and metastasis in HCC cell lines. (A) Western blot analysis of 9 HOX genes knockdown in HCCLM3 cells. (B) The invasion and migration rate of HCCLM3 cells with knockdown of the indicated genes.

Supplementary Figure S3

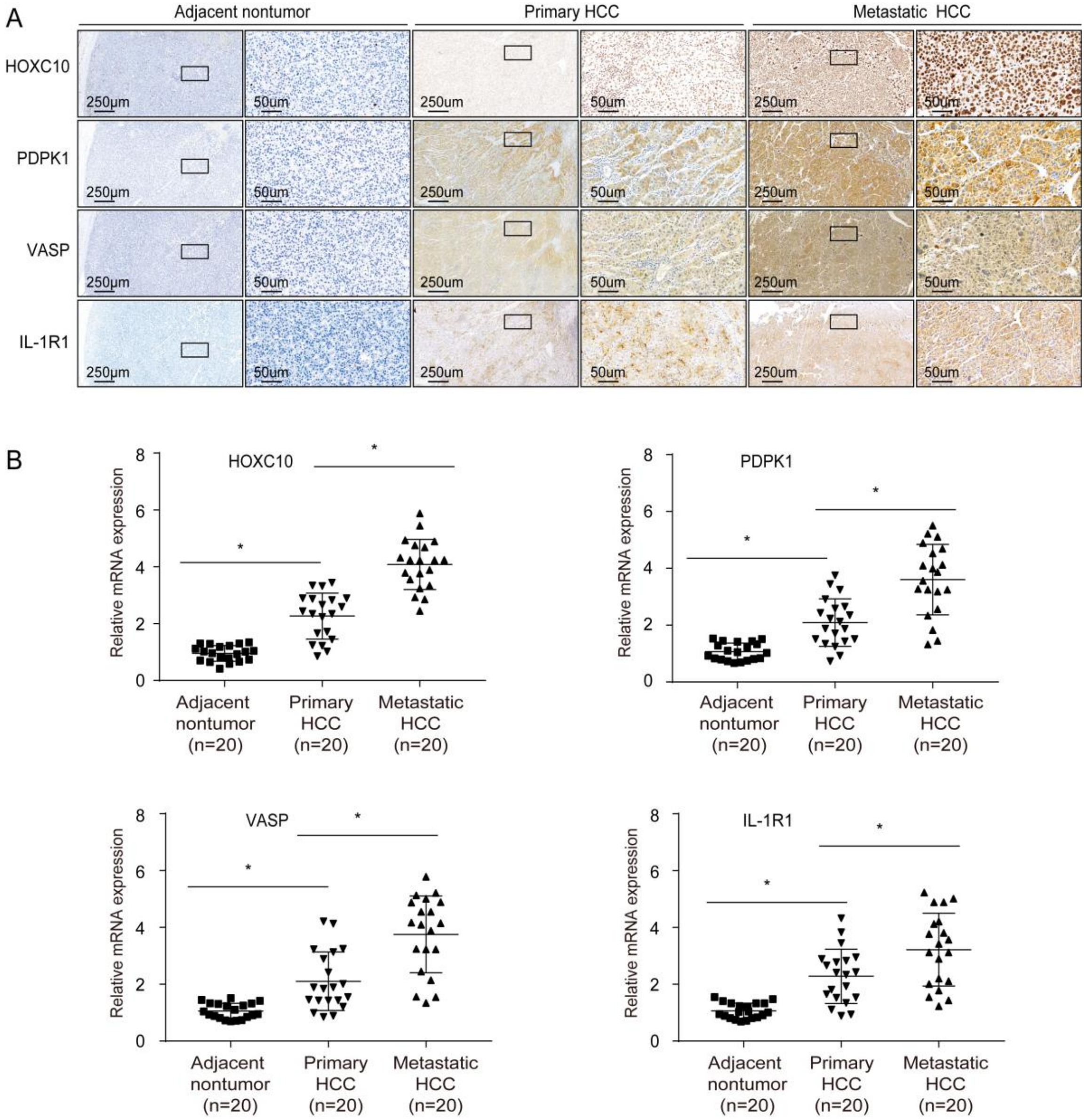

Supplementary Figure S3. The expression of HOXC10, PDPK1,VASP and IL-1R1 expression in 20 pairs of adjacent nontumorous tissues, primary HCC tissues and metastatic HCC tissues. (A) Immunohistochemistry was used to detect the expression of HOXC10, PDPK1, VASP and IL-1R1. The scale bars represent 250 μm (low magnification) and 50 μm (high magnification). (B) Real-time PCR were used to detect the HOXC10, PDPK1, VASP and IL-1R1 expression.

Supplementary Figure S4

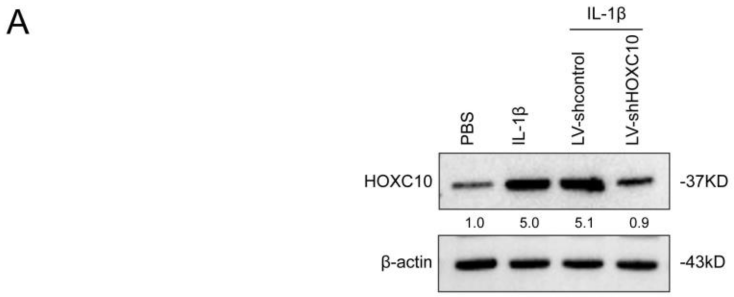

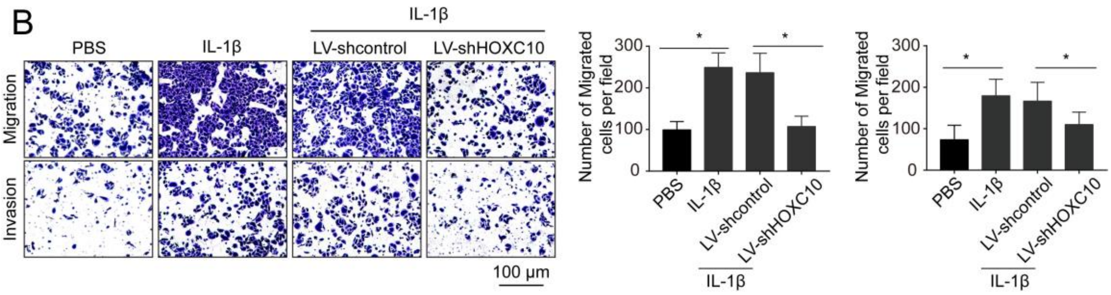

Supplementary Figure S4. HOXC10 is essential for IL-1β-induced HCC cell migration and invasion. Hep3B cells were infected with lentivirus LV-shHOXC10 or LV-shcontrol, and were treated with IL-1β (10ng/ml) for 24 hours. (A)Western blot analysis the expression of HOX genes. (B) Transwell assay was used to detect the migration and invasion abilities of the indicated HCC cells.

Supplementary Figure S5

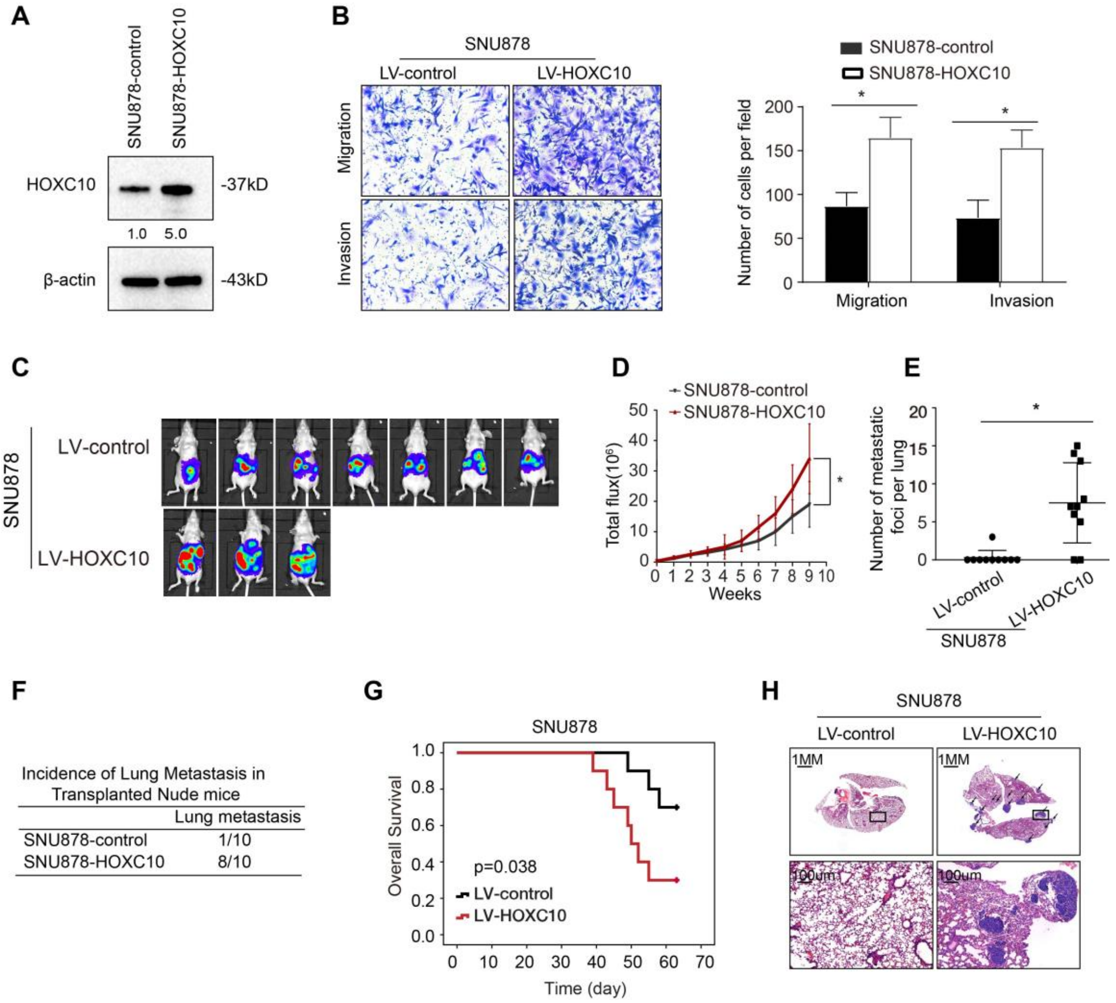

Supplementary Figure S5

(A) Western blotting analysis of HOXC10 overexpression in SNU878 cells.

(B) Transwell assay analysis of the migration and invasion abilities of the indicated HCC cells.

(C) The nude mice were divided into 2 groups (n=10 mice per group) and implanted with the indicated cells. BLI of the different groups is shown at 9 weeks following orthotopic implantation.

(D) The bioluminescent signals were recorded for 9 consecutive weeks after cell implantation.

(E) The number of lung metastatic foci in the lung was calculated.

(F) Incidence of lung metastasis in the transplanted nude mice.

(G) The overall survival times in each group are shown.

(H) Representative HE staining of lung tissues from the different groups is shown. The scale bars represent 1 mm (low magnification) and 100 μm (high magnification).

All the data are shown as the mean±s.d. * P<0.05 ** P<0.01.

Supplementary Figure S6

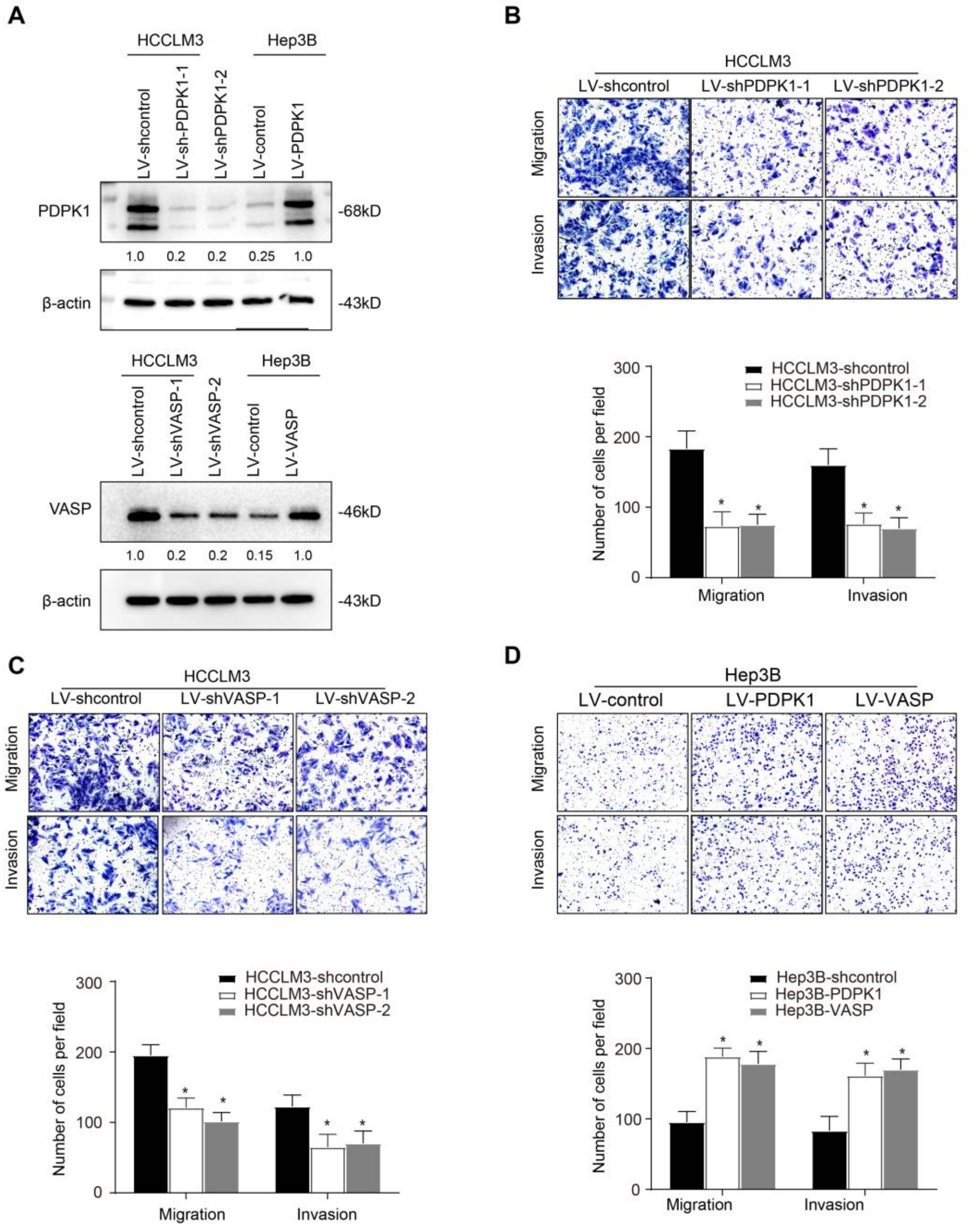

Supplementary Figure S6

(A) Western blotting analysis of PDPK1 and VASP expression in the indicated cell.

(B) Transwell assay showed knockdown PDPK1 decreased the migration and invasion abilities of the HCCLM3 cells.

(C) Transwell assay showed knockdown VASP decreased the migration and invasion abilities of the HCCLM3 cells.

(D) Transwell assay showed overexpression PDPK1 or VASP increased the migration and invasion abilities of the Hep3B cells.

Supplementary Figure S7

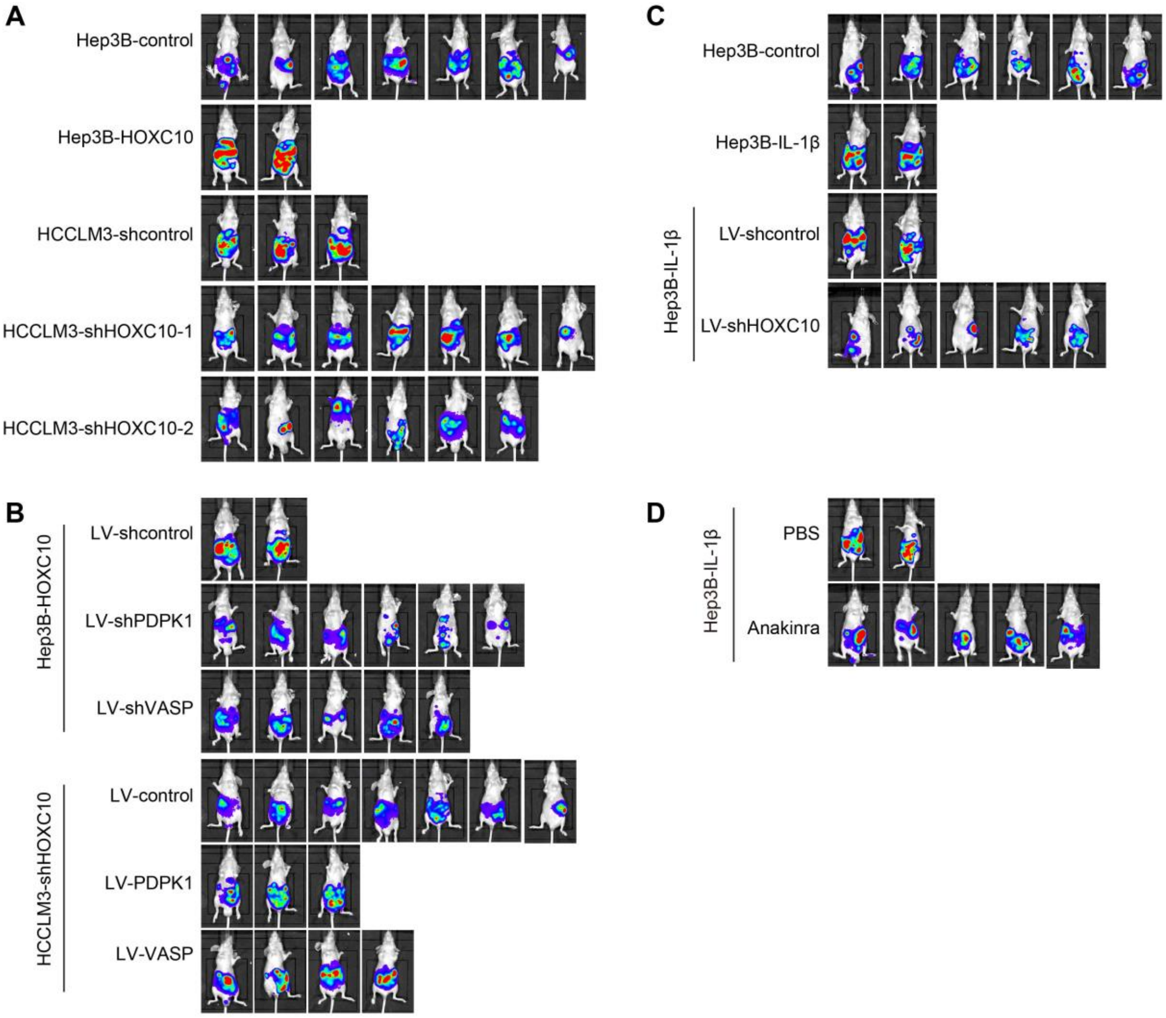

Supplementary Figure S7. The IVIS image

(A) The IVIS image of indicated group of Hep3B-control, Hep3B-HOXC10, HCCLM3-shcontrol, HCCLM3-shHOXC10-1and HCCLM3-shHOXC10-2 in Figure 1G.

(B) The IVIS image of indicated group, Hep3B-HOXC10-shcontrol, Hep3B-HOXC10-shPDPK1, Hep3B-HOXC10-shVASP, HCCLM3-shHOXC10-control, HCCLM3-shHOXC10-PDPK1 and HCCLM3-shHOXC10-VASP in Figure 3C.

(C) The IVIS image of indicated group, Hep3B-control, Hep3B-IL-1β，Hep3B-IL-1β-shcontrol and Hep3B-IL-1β-shHOXC10 in Figure 6C.

(D) The IVIS image of indicated group, Hep3B-IL-1β+PBS and Hep3B-IL-1β+Anakinra in Figure 6J.

Supplementary Figure S8

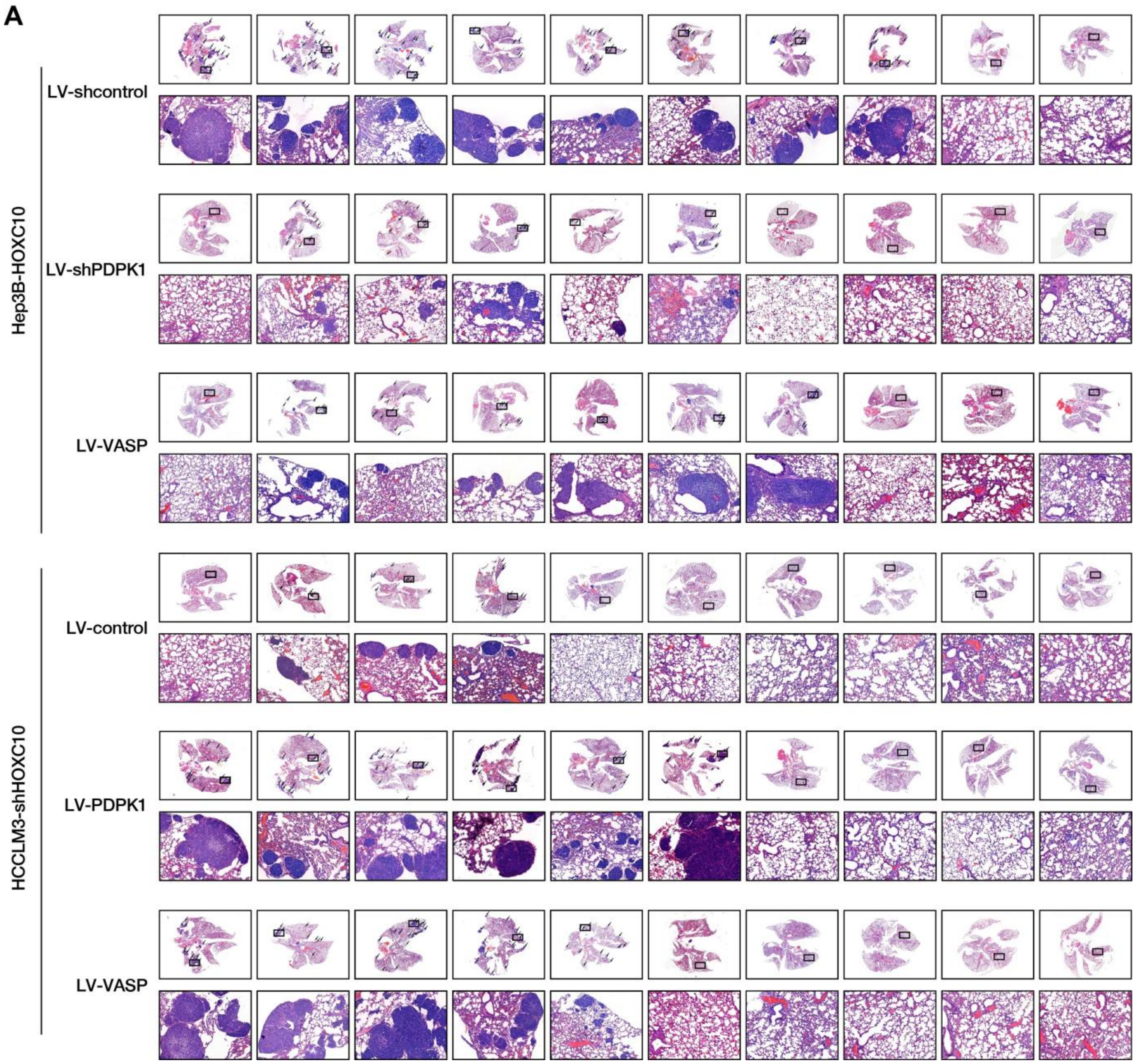

Supplementary Figure S8. The HE staining of lung tissues of indicated group, Hep3B-HOXC10-shcontrol, Hep3B-HOXC10-shPDPK1, Hep3B-HOXC10-shVASP, HCCLM3-shHOXC10-control, HCCLM3-shHOXC10-PDPK1, HCCLM3-shHOXC10-VASP in Figure 3H.

Supplementary Figure S9

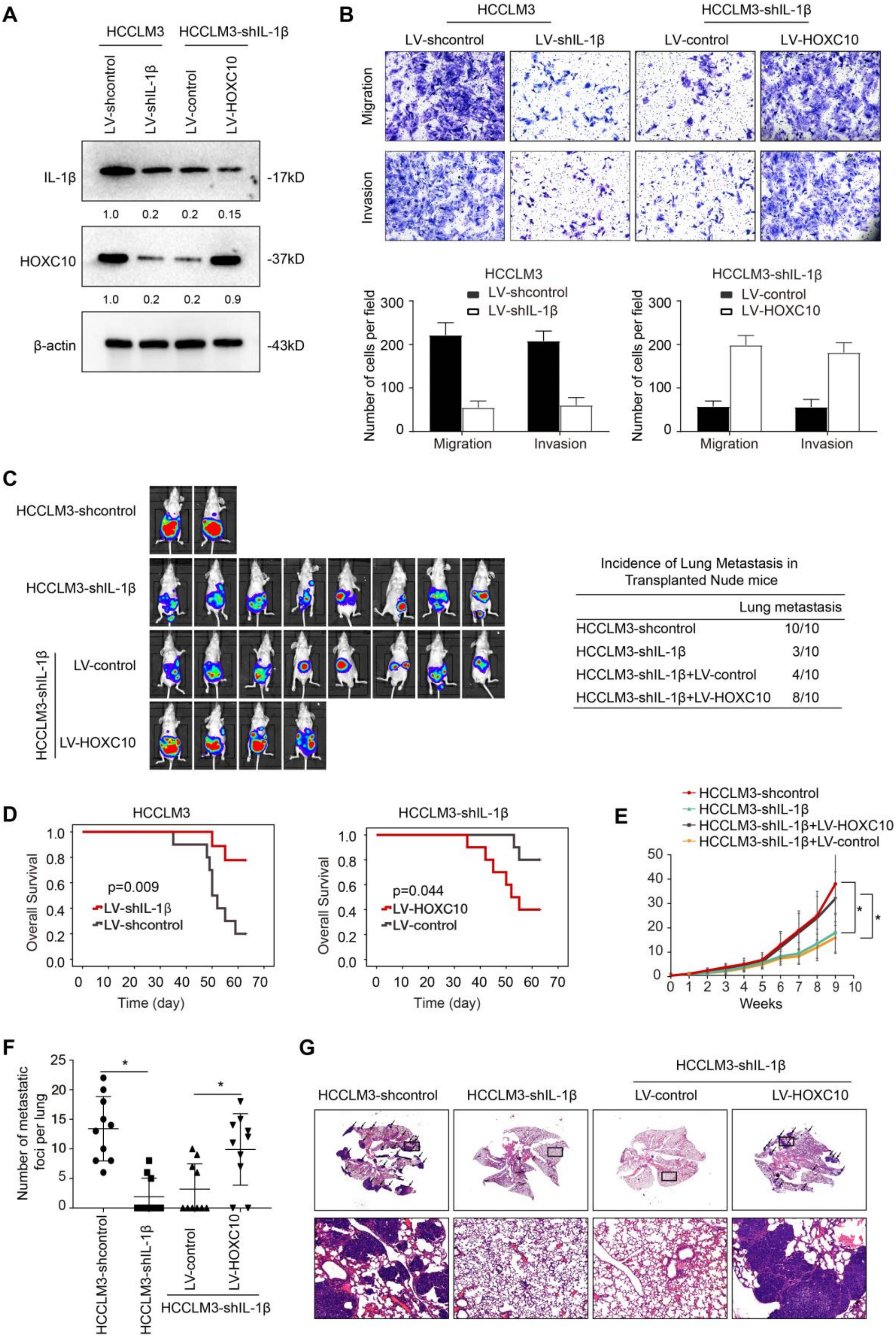

Supplementary Figure S9. HOXC10 is essential for IL-1β-mediated HCC metastasis.

(A) Western blot analysis showing HOXC10 and IL-1β expression in HCCLM3 after HCCLM3-shIL-1β cells were infected with lentivirus LV-HOXC10 or LV-control,

(B) Transwell assay analysis of the migration and invasion abilities of the indicated HCC cells

(C-G) *In vivo* metastasis assays. The indicated HCC cell lines were transplanted into the livers of nude mice. (C) Bioluminescent images and incidence of lung metastasis in transplanted nude mice. (D) Overall survival. (E) bioluminescence signals. (F) The number of lung metastatic foci in the lung was calculated. (G) Representative HE staining of lung tissues from the different groups is shown. The scale bars represent 1 mm (low magnification) and 100 μm (high magnification).

All the data are shown as the mean±s.d. * $P<0.05$ ** $P<0.01$.

Supplementary Table S1. List of genes differentially expressed in Hep3B-HOXC10 versus Hep3B-control cells using a Affymetrix PrimeView Human gene expression array

Symbol	Fold change	Description
AKT2	4.42	v-akt murine thymoma viral oncogene homolog 2
PDPK1	3.80	3-phosphoinositide dependent protein kinase-1
ACTA1	3.78	actin, alpha 1, skeletal muscle
MARK2	3.70	MAP/microtubule affinity-regulating kinase 2
LRRC8A	3.64	leucine rich repeat containing 8 family, member A
AKT1S1	3.22	AKT1 substrate 1 (proline-rich)
CASP2	3.12	caspase 2, apoptosis-related cysteine peptidase
BRD3	2.91	bromodomain containing 3
VASP	2.87	vasodilator-stimulated phosphoprotein
RPS6KB2	2.82	ribosomal protein S6 kinase, 70kDa, polypeptide 2
PODXL2	2.80	podocalyxin-like 2
KRT8	2.79	keratin 8
AKNA	2.73	AT-hook transcription factor
ZNF664	2.69	zinc finger protein 664
BMP6	2.69	bone morphogenetic protein 6
LAMP1	2.54	lysosomal-associated membrane protein 1
UBXN7	2.52	UBX domain protein 7
IER5L	2.52	immediate early response 5-like
PRRC2C	2.51	proline-rich coiled-coil 2C
TRIM2	2.44	tripartite motif containing 2
FLNA	2.44	filamin A, alpha
DAPK3	2.41	death-associated protein kinase 3
KIF1B	2.38	kinesin family member 1B
ITPRIPL2	2.37	inositol 1,4,5-trisphosphate receptor interacting protein-like 2
AKAP8L	2.36	A kinase (PRKA) anchor protein 8-like
JUP /// KRT17	2.33	junction plakoglobin /// keratin 17
GDF15	2.33	growth differentiation factor 15
GANAB	2.32	glucosidase, alpha; neutral AB
WIF1	2.30	WNT inhibitory factor 1
PTRF	2.24	polymerase I and transcript release factor
ANKRD11	2.21	ankyrin repeat domain 11
TAGLN	2.20	transgelin
P4HB	2.20	prolyl 4-hydroxylase, beta polypeptide
NEXN	2.19	nexilin (F actin binding protein)
EGFR	2.17	epidermal growth factor receptor

TSC22D3	2.16	TSC22 domain family, member 3
TNFRSF12A	2.16	tumor necrosis factor receptor superfamily, member 12A
GPATCH8	2.16	G patch domain containing 8
COL1A2	2.15	collagen, type I, alpha 2
RXRB	2.14	retinoid X receptor, beta
PAX6	2.13	paired box 6
NDST1	2.13	N-deacetylase/N-sulfotransferase (heparan glucosaminyl) 1
HIC2	2.13	hypermethylated in cancer 2
PYGB	2.13	phosphorylase, glycogen; brain
CNN2	2.12	calponin 2
SETD5	2.12	SET domain containing 5
CBX4	2.12	chromobox homolog 4
CLPTM1	2.11	cleft lip and palate associated transmembrane protein 1
HIST1H4A	2.11	histone cluster 1, H4a
TMEM158	2.10	transmembrane protein 158 (gene/pseudogene)
ZFP36L2	2.10	zinc finger protein 36, C3H type-like 2
RC3H2	2.10	ring finger and CCCH-type domains 2
MAP4	2.10	microtubule-associated protein 4
EIF4G1	2.08	eukaryotic translation initiation factor 4 gamma, 1
SRSF6	2.08	serine/arginine-rich splicing factor 6
RUFY3	2.06	RUN and FYVE domain containing 3
CDH19	2.06	cadherin 19, type 2
LTBP3	2.05	latent transforming growth factor beta binding protein 3
NAP1L2	2.04	nucleosome assembly protein 1-like 2
FHL2	2.03	four and a half LIM domains 2
MAZ	2.02	MYC-associated zinc finger protein (purine-binding transcription factor)
HIST1H4Ac	2.02	Histone cluster 1, H4a
MCM4	2.02	minichromosome maintenance complex component 4
CDC42SE1	2.01	CDC42 small effector 1
DHFR	2.00	dihydrofolate reductase
ATF5	2.00	activating transcription factor 5
NCS1	2.00	neuronal calcium sensor 1
RPS6KB2	2.00	ribosomal protein S6 kinase, 70kDa, polypeptide 2
NEDD4	-2.01	neural precursor cell expressed, developmentally down-regulated 4, E3 ubiquitin protein ligase
HABP2	-2.01	hyaluronan binding protein 2
CXCL1	-2.03	chemokine (C-X-C motif) ligand 1 (melanoma growth

		stimulating activity, alpha)
TTC6	-2.03	tetratricopeptide repeat domain 6
BIRC3	-2.03	baculoviral IAP repeat containing 3
PLK4	-2.04	polo-like kinase 4
ANGPTL3	-2.04	angiopoietin-like 3
ITIH2	-2.04	inter-alpha-trypsin inhibitor heavy chain 2
DLK1	-2.04	delta-like 1 homolog (Drosophila)
HS6ST2	-2.05	heparan sulfate 6-O-sulfotransferase 2
F2	-2.07	coagulation factor II (thrombin)
SACM1L	-2.07	SAC1 suppressor of actin mutations 1-like (yeast)
SLC1A3	-2.08	solute carrier family 1 (glial high affinity glutamate transporter), member 3
FRAS1	-2.08	Fraser syndrome 1
ASPH	-2.08	aspartate beta-hydroxylase
DYRK1A	-2.09	dual-specificity tyrosine-(Y)-phosphorylation regulated kinase 1A
MBNL3	-2.09	muscleblind-like splicing regulator 3
MAN1A1	-2.10	mannosidase, alpha, class 1A, member 1
MTUS1	-2.11	microtubule associated tumor suppressor 1
ALCAM	-2.12	activated leukocyte cell adhesion molecule
TFRC	-2.13	transferrin receptor (p90, CD71)
PAH	-2.13	phenylalanine hydroxylase
ADH6	-2.13	alcohol dehydrogenase 6 (class V)
CHODL	-2.13	chondrolectin
PLA1A	-2.14	phospholipase A1 member A
FGB	-2.14	fibrinogen beta chain
UGT2B4	-2.14	UDP glucuronosyltransferase 2 family, polypeptide B4
CXCL5 /// GLYR1	-2.15	chemokine (C-X-C motif) ligand 5 /// glyoxylate reductase 1 homolog (Arabidopsis)
NF1	-2.16	neurofibromin 1
BDH1	-2.17	3-hydroxybutyrate dehydrogenase, type 1
HLF	-2.18	hepatic leukemia factor
MAP3K8	-2.18	mitogen-activated protein kinase kinase kinase 8
MAN1A1	-2.18	mannosidase, alpha, class 1A, member 1
GJA1	-2.19	gap junction protein, alpha 1, 43kDa
YAP1	-2.19	Yes-associated protein 1
ZNF518A	-2.19	zinc finger protein 518A
SLC6A14	-2.19	solute carrier family 6 (amino acid transporter), member 14

AKR1B15	-2.20	aldo-keto reductase family 1, member B15
NAPEPLD	-2.21	N-acyl phosphatidylethanolamine phospholipase D
DLG1	-2.21	discs, large homolog 1 (Drosophila)
LTB	-2.21	lymphotoxin beta (TNF superfamily, member 3)
MET	-2.23	met proto-oncogene (hepatocyte growth factor receptor)
HCAR2 /// HCAR3	-2.23	hydroxycarboxylic acid receptor 2 /// hydroxycarboxylic acid receptor 3
VSNL1	-2.23	visinin-like 1
KNG1	-2.25	kininogen 1
SCG5	-2.25	secretogranin V (7B2 protein)
UGT2B4	-2.28	UDP glucuronosyltransferase 2 family, polypeptide B4
TNFSF4	-2.29	tumor necrosis factor (ligand) superfamily, member 4
LOC100509445 /// OVOS2	-2.30	uncharacterized LOC100509445 /// ovostatin 2
C1orf64	-2.31	chromosome 1 open reading frame 64
SAA2-SAA4 /// SAA4	-2.33	SAA2-SAA4 readthrough /// serum amyloid A4, constitutive
VNN2	-2.34	vanin 2
CCL20	-2.35	chemokine (C-C motif) ligand 20
ALDH8A1	-2.35	aldehyde dehydrogenase 8 family, member A1
CACNB2	-2.35	calcium channel, voltage-dependent, beta 2 subunit
SLC22A3	-2.36	solute carrier family 22 (extraneuronal monoamine transporter), member 3
CXCL2	-2.38	chemokine (C-X-C motif) ligand 2
KRT23	-2.38	keratin 23 (histone deacetylase inducible)
MCC	-2.38	mutated in colorectal cancers
MCTP1	-2.39	multiple C2 domains, transmembrane 1
PHF6	-2.39	PHD finger protein 6
KIAA0754	-2.41	KIAA0754
ACSL4	-2.42	acyl-CoA synthetase long-chain family member 4
FAM176A	-2.42	family with sequence similarity 176, member A
HAL	-2.42	histidine ammonia-lyase
FBXL17	-2.42	F-box and leucine-rich repeat protein 17
ADH4	-2.43	alcohol dehydrogenase 4 (class II), pi polypeptide
ANXA10	-2.52	annexin A10
ZNF512	-2.52	zinc finger protein 512
VAV3	-2.54	vav 3 guanine nucleotide exchange factor
C19orf80	-2.54	chromosome 19 open reading frame 80

NR1H4	-2.55	nuclear receptor subfamily 1, group H, member 4
UGT2A3	-2.56	UDP glucuronosyltransferase 2 family, polypeptide A3
QSER1	-2.56	glutamine and serine rich 1
GPX2	-2.61	glutathione peroxidase 2 (gastrointestinal)
HLF	-2.62	hepatic leukemia factor
DEFB1	-2.64	defensin, beta 1
LYZ	-2.65	lysozyme
NT5E	-2.66	5'-nucleotidase, ecto (CD73)
CGA	-2.76	glycoprotein hormones, alpha polypeptide
AKR1B10	-2.77	aldo-keto reductase family 1, member B10 (aldose reductase)
NT5E	-2.78	5'-nucleotidase, ecto (CD73)
AKR1B10 /// AKR1B15	-2.79	aldo-keto reductase family 1, member B10 (aldose reductase) /// aldo-keto reductase family 1, member B15
CPB2	-2.80	carboxypeptidase B2 (plasma)
PAH	-2.82	phenylalanine hydroxylase
VNN3	-2.82	vanin 3
TDO2	-2.85	tryptophan 2,3-dioxygenase
TRUB1	-2.90	TruB pseudouridine (psi) synthase homolog 1 (E. coli)
ZPLD1	-2.90	zona pellucida-like domain containing 1
UBE2E1	-2.92	Ubiquitin-conjugating enzyme E2E 1
C4BPB	-2.93	complement component 4 binding protein, beta
LINC00597	-3.04	long intergenic non-protein coding RNA 597
SRRM2	-3.04	serine/arginine repetitive matrix 2
GPAM	-3.07	glycerol-3-phosphate acyltransferase, mitochondrial
CXCL10	-3.13	chemokine (C-X-C motif) ligand 10
CXCL3	-3.16	chemokine (C-X-C motif) ligand 3
SULT2A1	-3.45	sulfotransferase family, cytosolic, 2A, dehydroepiandrosterone (DHEA)-preferring, member 1
ATP2B2	-3.64	ATPase, Ca++ transporting, plasma membrane 2
C4BPA	-3.86	complement component 4 binding protein, alpha

Supplementary Table S2. Correlation between PDPK1 expression and clinicopathological characteristics of HCCs in two independent cohorts of human HCC tissues

Clinicopathological variables		Cohort I Tumor PDPK1 expression Negative (n=224)	Cohort I Tumor PDPK1 expression Positive (n=173)	*P* Value	Cohort II Tumor PDPK1expression Negative (n=204)	Cohort II Tumor PDPK1expression Positive (n=121)	*P* Value
Age	≤50	93	65	0.426	36	28	0.229
	>50	131	108		168	93	
Sex	female	52	33	0.319	33	14	0.254
	male	172	140		171	107	
Serum AFP	≤20ng/ml	170	128	0.664	62	35	0.780
	>20ng/ml	54	45		142	86	
Virus infection	HBV	166	116	0.347	148	84	0.816
	HCV	16	18		16	8	
	HBV+HCV	12	8		11	8	
	None	30	31		29	21	
Cirrrhosis	absent	75	42	0.046	56	22	0.059
	present	149	131		148	99	
Child-pugh score	Class A	168	138	0.262	169	93	0.187
	Class B	56	35		35	28	
Tumor number	single	183	131	0.147	152	76	0.026
	multiple	41	42		52	45	
Maximal tumor size	≤5cm	142	71	<0.001*	105	40	0.001
	>5cm	82	102		99	81	
Tumor encapsulation	absent	102	59	0.021	67	57	0.010
	present	122	114		137	64	
Microvascular invasion	absent	126	79	0.036	127	67	0.221
	present	98	94		77	54	
Tumor differentiation	I-II	146	119	0.449	145	83	0.636
	III-IV	78	54		59	38	
TNM stage	I-II	156	94	0.002	154	72	0.002
	III	68	79		50	49	

Supplementary Table S3. Correlation between VASP expression and clinicopathological characteristics of HCCs in two independent cohorts of human HCC tissues

Clinicopathological variables		Cohort I Tumor VASP expression			Cohort II Tumor VASP expression		
		Negative (n=249)	Positive (n=148)	*P* Value	Negative (n=185)	Positive (n=140)	*P* Value
Age	⩽50	94	64	0.294	43	21	0.064
	>50	154	84		142	119	
Sex	female	59	26	0.150	32	15	0.095
	male	190	122		153	125	
Serum AFP	≤20ng/ml	186	112	0.828	57	40	0.662
	>20ng/ml	63	36		128	100	
Virus infection	HBV	174	108	0.365	127	105	0.011
	HCV	19	15		18	6	
	HBV+HCV	12	8		6	13	
	None	44	17		34	16	
Cirrrhosis	absent	73	44	0.931	47	31	0.495
	present	176	104		138	109	
Child-pugh score	Class A	192	114	0.985	144	118	0.145
	Class B	57	34		41	22	
Tumor number	single	201	113	0.300	133	95	0.431
	multiple	48	35		52	45	
Maximal tumor size	≤5cm	149	64	0.001	94	51	0.010
	>5cm	100	84		91	89	
Tumor encapsulation	absent	108	53	0.138	59	65	0.008
	present	141	95		126	75	
Microvascular invasion	absent	142	63	0.005	120	74	0.029
	present	107	85		65	66	
Tumor differentiation	I-II	160	105	0.171	135	93	0.202
	III-IV	89	43		50	47	
TNM stage	I-II	172	78	0.001	140	86	0.006
	III	77	70		45	54	

Supplementary Table S4. Correlation between IL-1R1expression and clinicopathological characteristics of HCCs in two independent cohorts of human HCC tissues

Clinicopathological variables		Cohort I Tumor IL-1R1 expression Negative (n=272)	Cohort I Tumor IL-1R1 expression Positive (n=125)	*P* Value	Cohort II Tumor IL-1R1 expression Negative (n=219)	Cohort II Tumor IL-1R1 expression Positive (n=106)	*P* Value
Age	≤50	115	43	0.136	46	18	0.393
	>50	157	82		173	88	
Sex	female	60	25	0.642	34	13	0.433
	male	212	100		185	93	
Serum AFP	≤20ng/ml	208	90	0.339	71	26	0.145
	>20ng/ml	64	35		148	80	
Virus infection	HBV	200	82	0.078	157	75	0.892
	HCV	22	12		16	8	
	HBV+HCV	16	4		14	5	
	None	34	27		32	18	
Cirrrhosis	absent	76	41	0.324	53	25	0.903
	present	196	84		166	81	
Child-pugh score	Class A	205	101	0.232	176	86	0.870
	Class B	67	24		43	20	
Tumor number	single	232	82	0.000	157	71	0.385
	multiple	40	43		62	35	
Maximal tumor size	≤5cm	165	48	0.000	110	35	0.003
	>5cm	107	77		109	71	
Tumor encapsulation	absent	129	32	0.000	73	51	0.010
	present	143	93		146	55	
Microvascular invasion	absent	157	48	0.000	142	52	0.007
	present	115	77		77	54	
Tumor differentiation	I-II	179	86	0.557	156	72	0.541
	III-IV	93	39		63	34	
TNM stage	I-II	202	48	0.000	164	62	0.003
	III	70	77		55	44	

Supplementary Table S5. Primer sequences used in the study

Primer name	Primer sequences	Enzyme
**Primers for PDPK1 promoter construct:**		
(-2053/+75) PDPK sense:	5'- TATAGGTACCTTCCAGACCCGCAGGAGC -3'	KpnI,
(-1302/+75) PDPK sense:	5'- TATAGGTACCCACTGTGTACTAGTTCCT -3'	KpnI,
(-694/+75) PDPK sense:	5'- TATAGGTACCCAGTGAGCAGAGATCGAG -3'	KpnI,
(-401/+75) PDPK sense:	5'- TATAGGTACCGCTTGTTAGTTGTGAAA -3'	KpnI,
antisense:	5'- ATATCTCGAG TCAGCGTCCTCCTCCCCG -3'	XhoI
**Primers for PDPK1 promoter site-directed mutagenesis:**		
HOXC10 binding site 4 mutation sense:	5'- TACACAGCAGTcgccAACTAATACAC -3'	
HOXC10binding site 4mutation antisense:	5'- CAACTTTATTTTgccgTTATACTGACAC -3'	
HOXC10binding site 3 mutation sense:	5'- GTGTCAGTATAccgcAAAATAAAGTT -3'	
HOXC10 binding site 3mutation antisense:	5'- AACTTTATTTTgcggTATACTGACAC -3'	
HOXC10 binding site 2 mutation sense:	5'- ATTATCGACATcgcaAACTACAAGGC -3'	
HOXC10binding site 2 mutation antisense:	5'- GCCTTGTAGTTtgcgATGTCGATAAT -3'	
HOXC10 binding site 1 mutation sense:	5'- GCCCGGCTAATTtcgcATTTTTAGTAG -3'	
HOXC10 binding site 1 mutation antisense:	5'- CTTTGCTAGTTctgtAACTAACAAGC -3'	
**Primers used for ChIP in the PDPK1 promoter:**		
Distant region sense:	5'- AGGAAGTGAATCTTTCGG-3'	
Distant region antisense:	5'- CTCAGGCTCTCTTGTGAA -3'	
HOXC10 binding site 4 sense:	5'- GGCCTCTCCTGGATTCCA -3'	
HOXC10binding site 4 antisense:	5'- ACGCACATGAGGCAAGCT -3'	
HOXC10binding site 3 sense:	5'- GTCCTTCACTGCTTCCGA -3'	
HOXC10binding site 3 antisense:	5'- GGAGATTGAGACCACGGT -3'	
HOXC10 binding site 2/1 sense:	5'- AGTCCCAGCTACTCGGGA -3'	
HOXC10binding site 2/1antisense:	5'- GGATCACTTGAGGTGAGG -3'	
**Primers for VASP promoter construct**:		
(-1915/+69)VASP sense:	5'- TATAGGTACCTCCACACCTCCTTAAGTT -3'	KpnI,
(−1555/+69)VASP sense:	5'- TATAGGTACCGTGCTGGGATTACAGGCG -3'	KpnI,
(- 928/+69)VASP sense:	5'-TATAGGTACCAGCTGCTGAGCCGGGCGGG-3'	KpnI,
(- 398/+69)VASP sense:	5'-TATAGGTACCACCGAGGAACAGGGTTCAA-3'	KpnI,
antisense:	5'- ATATCTCGAG CCAAATCTTCCTGGAGGG -3'	XhoI
**Primers for VASP promoter site-directed mutagenesis**:		
HOXC10 binding site 3 mutation sense:	5’- GTTTAATTTTTcggaAGGAGTCTTG - 3’	
HOXC10 binding site 3 mutation antisense:	5'- CAAGACTCCTtccgAAAAATTAAAC -3'	
HOXC10binding site 2 mutation sense:	5'- TCACTGCTACCacgcAGATCGTTATG -3'	
HOXC10binding site 2 mutation antisense:	5'- CATAACGATCTgcgtGGTAGCAGTGA -3'	
HOXC10 binding site 1 mutation sense:	5'- CACCTGAGGTCgcgcGTTTGAGACCA -3'	
HOXC10binding site 1 mutation antisense:	5'- TGGTCTCAAACgcgcGACCTCAGGTG -3'	
**Primers used for ChIP in the VASP promoter:**		
Distant region sense:	5'- CCCTGACCTCAAGTGATC -3'	
Distant region antisense:	5'- CTGAAGCACGGAGTGAGC -3'	
HOXC10 binding site 3 sense:	5'- CTATTCCCTGCCCTCATC -3'	

HOXC10binding site 3 antisense:	5'- ACACCTGTAATCCCAGCT -3'	
HOXC10 binding site 2 sense:	5'- CTATTCCCTGCCCTCATC -3'	
HOXC10binding site 2 antisense:	5'- TATGCTAGACATAGGGTC -3'	
HOXC10 binding site 1 sense:	5'- GAGAGGAGAACACAGTTA -3'	
HOXC10 binding site 1 antisense:	5'- CTGGAGGGCGCTGGCTCG -3'	
**Primers for HOXC10 promoter construct:**		
(-1651/+146)HOXC10 sense:	5'- TATAGGTACCAAATGGCTCCCTGGATTG -3'	KpnI
(-980/+146)FGFBP1 sense:	5'- TATAGGTACCCCGTGTGATACGTGACTCC -3'	KpnI
(-320/+146)FGFBP1 sense:	5'- TATAGGTACCACTGTTTTGAGCCCCGGGT -3'	KpnI
(+60/+146)FGFBP1 sense:	5'- TATAGGTACCTGTGGATGTGTGTGTTTT -3'	KpnI
antisense:	5'- ATATCTCGAGTAACGTTGATTTAAATAT -3'	XhoI
**Primers for HOXC10 promoter site-directed mutagenesis:**		
c-Jun binding site 1 mutation sense:	5'- GATGGTGcataAGGAGC -3'	
c-Jun binding site 1 mutation antisense:	5'- GCTCCTtatgCACCATC-3'	
SP1 binding site 1 mutation sense:	5'- GTGACCCaactCCTGGCC -3'	
SP1 binding site1 mutation antisense:	5'- GGCCAGGagttGGGTCAC -3'	
NF-kB binding site1 mutation sense:	5'- GGCTGGAGtacgCCAGTC -3'	
NF-kB binding site1 mutation antisense:	5'- GACTGGcgtaCTCCAGCC-3'	
c-Jun binding site2 mutation sense:	5'- GTACCTGgccaGTGTCTC-3'	
c-Jun binding site2 mutation antisense:	5'- GAGACAtggcCAGGTAC-3'	
**Primers used for ChIP in the c-Jun promoter:**		
Distant region sense:	5'- CCAAGTCTGGGGCCTAAA -3'	
Distant region antisense:	5'- ATCCCCGACCCGAGAGCT -3'	
c-Jun binding site 1 sense:	5'-AGCTCTCGGGTCGGGGAT-3'	
c-Jun binding site 1 antisense:	5'- ATCCCCGACCCGAGAGCT -3'	
c-Jun binding site 2 sense:	5'-GCTCATCAGATCCCCCCAA-3'	
c-Jun binding site 2 antisense:	5'-GCTGGTTACTGGATAATG -3'	
**Primers for real-time PCR:**		
GAPDH sense:	5’-GCACCGTCAAGGCTGAGAAC-3’	
GAPDH antisense:	5’-TGGTGAAGACGCCAGTGGA-3’	
PDPK1 sense	5’-GGAACAGCGCAGTACGTTTCT-3’	
PDPK1 antisense	5’-CTCGTTTCCAGCTCGGAATGG-3’	
VASP sense:	5’-ATGGCAACAAGCGATGGCT-3’	
VASP antisense:	5’-CGATGGCACAGTTGATGACCA-3’	
HOXA1 sense	5'- CGCTCCCGCTGTTTACTC-3'	
HOXA1 antisense	5'-AGGCTCTGGTGCTCCTGTCC-3'	
HOXA2 sense	5'- AGCAGCAGGCTCCCAATG-3'	
HOXA2 antisense	5'-GGGAACCTGGCAAACTGG-3'	
HOXA3 sense	5'-CCAGCCCTCTTTGGTCTAACTC-3'	
HOXA3 antisense	5'- AGCTTGGGTGCTTCCTGAAT-3'	
HOXA4 sense	5'- CGGAGGATGAAGTGGAAGAAAG-3'	
HOXA4 antisense	5'- TGGAGGAGGGAACGGGTGT-3'	
HOXA5 sense	5'- TTCAACCGTTACCTGACCCG-3'	
HOXA5 antisense	5'-CGGCCATGCTCATGCTTT-3'	

HOXA6 sense	5'- CTCGGGCAGTGGCAAGCAGA-3'
HOXA6 antisense	5'- GTGGGATTCACAAAATAGGAACTCA-3'
HOXA7 sense	5'- CCCTGGATGCGGTCTTCA-3'
HOXA7 antisense	5'- CCTTCGTCCTTATGCTCTTTCT-3'
HOXA9 sense	5'- AAGGCGACGGTGTTTGGC-3'
HOXA9 antisense	5'- CCGACAGCGGTTCAGGTT-3'
HOXA10 sense	5'- GGCTCACGGCAAAGAGTGG-3'
HOXA10 antisense	5'- CTTCAGTTTCATCCTGCGGTTC-3'
HOXA11 sense	5'-TACTCCTACTCCTCCAACCTGC -3'
HOXA11 antisense	5'-TCCTGCCCACGGTGCTAT-3'
HOXA13 sense	5'- CGACGTGGTCTCCCATCCCT-3'
HOXA13 antisense	5'- CGTGGCGTATTCCCGTTCA-3'
HOXB1 sense	5'- GGCTTGTCCGATGGCTAC-3'
HOXB1 antisense	5'- CTGTCTTGGGTGGGTTTCTC-3'
HOXB2 sense	5'- ACCGAAAGGCAGGTCAAAGT-3'
HOXB2 antisense	5'-CTCTAAGCGAACGGCTAAAGG-3'
HOXB3 sense	5'- ATGCAGGGCAGTCCGGTGTA-3'
HOXB3 antisense	5'- GGTGATGGGAAAGGTGGTTG-3'
HOXB4 sense	5'-CAGGTCTTGGAGCTGGAGAAGG-3'
HOXB4 antisense	5'-CGAGCGGATCTTGGTGTTGG-3'
HOXB5 sense	5'-CGGCTACAATTACAATGGGATG-3'
HOXB5 antisense	5'- GGCCTCGTCTATTTCGGTGA-3'
HOXB6 sense	5'-GGCGAGACAGAAGAGCAGAA-3'
HOXB6 antisense	5'-CGTCAGGTAGCGATTGTAGTGA-3'
HOXB7 sense	5'-CAGAGGGACTCGGACTTGG-3'
HOXB7 antisense	5'- GTCTGGTAGCGGGTGTAGGT-3'
HOXB8 sense	5'- TTATTATGACTGCGGCTTCG-3'
HOXB8 antisense	5'- GGGTTCTGCTGGTAGGGAG-3'
HOXB9 sense	5'- TGTCTGAGAATGTCCATTTCTGGGA-3'
HOXB9 antisense	5'- TCCCAGAAATGGACATTCTCAGACA-3'
HOXB13 sense	5'-CCCGTGCCTTATGGTTACTT-3'
HOXB13 antisense	5'- GGACCTGGTGGGTTCTGTT-3'
HOXC4 sense	5'- CCAGCAAGCAACCCATAGT -3'
HOXC4 antisense	5'- GGTGGTCCTTCTTCCATTTCA -3'
HOXC5 sense	5'- TACAGTCAGAAGGCGGCTCG -3'
HOXC5 antisense	5'- GTGGCTCATGTGCAGTTTGGTC-3'
HOXC6 sense	5'- TTCCTACTTCACTAACCCTTCC-3'
HOXC6 antisense	5'- TCTCCTGTGGCGAATAAAA-3'
HOXC8 sense	5'- GAACCCGTGCTCGCTTAG-3'
HOXC8 antisense	5'- ACGCTCGCCTCTTGCTGA-3'
HOXC9 sense	5'- CCGCTCCACGAGGAAGAA-3'
HOXC9 antisense	5'- GCCGCTCGGTGAGATTGA-3'
HOXC10 sense	5'- CAGCCCAGACACCTCGGATA -3'
HOXC10 antisense	5'- CAATTCCAGCGTCTGGTGTTTAG -3'

HOXC11 sense	5'- GCCTCCAACCTCTATCTGCC-3'
HOXC11 antisense	5'- TGTTCCGATGGTGCCACTT-3'
HOXC12 sense	5'- CCTTCGCTGTCCTACCCACG-3'
HOXC12 antisense	5'- AACCCTTGCCGTCCTCCAC-3'
HOXC13 sense	5'- CTTCCCAGACGTGGTTCCC-3'
HOXC13 antisense	5'- AGATGAGGCGCTTTCGATTT-3'
HOXD1 sense	5'- CTTCCCAGACGTGGTTCCC-3'
HOXD1 antisense	5'- GAGGCGCTTTCGATTTGC-3'
HOXD3 sense	5'-TTCGCCAAATCACAGCCCAATA-3'
HOXD3 antisense	5'- AGTTGCCGCCCACGTACACC-3'
HOXD4 sense	5'- CCGTGCGAGGAGTATTTGC-3'
HOXD4 antisense	5'- GGGTCGGACTGACTGTAGGC-3'
HOXD8 sense	5'- TACGACCCAGCAAGAGGC-3'
HOXD8 antisense	5'- CACGGAAACATTTGAGAAGG-3'
HOXD9 sense	5'-CGCTGTCCCTACACCAAATACC-3'
HOXD9 antisense	5'- CGCCGGGTCAGTCTCCTTT-3'
HOXD10 sense	5'- CCACCAACATTAAGGAAGA-3'
HOXD10 antisense	5'-GACAAGACTCAGGGACCAG-3'
HOXD11 sense	5'-TTTGACGAGTGCGGCCAGAG-3'
HOXD11 antisense	5'- GACGACGGTTGGGAAAGGAA-3'
HOXD12 sense	5'- CTATGTGGGCTCGCTTCTGA-3'
HOXD12 antisense	5'- GTCCGTCTTTGGCTGTTGG-3'
HOXD13 sense	5'- CGTCGTCCTCTTCTGCCGTTGT-3'
HOXD13 antisense	5'-AGGCGTGCGGCGATGACTT-3'

Supplementary Table S6. shRNA sequences used in the study

Gene name	shRNA sequences
HOXA3	5'-CCGGCCATCCTTCTCAGGGAAGAATCTCGAGATTCTTCCCTGAGAAGGATGGTTTTTG -3'
HOXA6	5'-CCGGCTCTACCAGGCTGGCTATGACCTCGAGGTCATAGCCAGCCTGGTAGAGTTTTTTG -3'
HOXA10	5'-CCGGCCCTATCTTGTGAAGTTGTTTCTCGAGAAACAACTTCACAAGATAGGGTTTTT-3'
HOXA13	5'-CCGGTCGCGGACAAGTACATGGATACTCGAGTATCCATGTACTTGTCCGCGATTTTT-3'
HOXB5	5'-CCGGGAAGAAGGACAACAAATTGAACTCGAGTTCAATTTGTTGTCCTTCTTCTTTTTTG-3'
HOXB7	5'-CCGGATGCGAAGCTCAGGAACTGACCTCGAGGTCAGTTCCTGAGCTTCGCATTTTTTG-3'
HOXC4	5'-CCGGCTGAACACAGTCCGGAATATTCTCGAGAATATTCCGGACTGTGTTCAGTTTTTTG -3'
HOXC10-1	5'-CCGGCTGGAGATTAGCAAGACCATTCTCGAGAATGGTCTTGCTAATCTCCAGTTTTTG-3'
HOXC10-2	5'- CCGGCTTACAGACAGACAAGTCAAACTCGAGTTTGACTTGTCTGTCTGTAAGTTTTTG-3'
HOXC10-3	5'- CCGGACCTAGTGTCAAGGAGGAGAACTCGAGTTCTCCTCCTTGACACTAGGTTTTTTG-3'
HOXD9	5'-CCGGCCGCCGAGTTCGCCTCGTGTACTCGAGTACACGAGGCGAACTCGGCGGTTTTT-3'
PDPK1-1	5'-CCGGATAAGCGGAAGGGTTTATTTCTCGAGAAATAAACCCTTCCGCTTATCTTTTTG-3'
PDPK1-2	5'- CCGGGCAGCAACATAGAGCAGTACACTCGAGTGTACTGCTCTATGTTGCTGCTTTTT-3'
VASP-1	5'-CCGGCGGGCCACTGTGATGCTTTATCTCGAGATAAAGCATCACAGTGGCCCGTTTTTG-3'
VASP-2	5'-CCGGCCAAGGATGAAGTCGTCTTCTCTCGAGAGAAGACGACTTCATCCTTGGTTTTTG-3'
IL-1β	5'- CCGGCTGACTTCACCATGCAATTTGCTCGAGCAAATTGCATGGTGAAGTCAGTTTTTG-3'
